# Viral communities associated with healthy and bleaching corals

**DOI:** 10.1111/j.1462-2920.2008.01652.x

**Published:** 2008-09

**Authors:** Kristen L Marhaver, Robert A Edwards, Forest Rohwer

**Affiliations:** 1 Center for Marine Biodiversity and Conservation, Scripps Institution of Oceanography, University of CaliforniaSan Diego, 9500 Gilman Dr, La Jolla, CA 92093-0202, USA; 2 Department of Computer Science, San Diego State University5500 Campanile Dr, San Diego, CA 92182, USA; 3 Department of Biology and Center for Microbial Sciences, San Diego State University5500 Campanile Dr, San Diego, CA 92182, USA

## Abstract

The coral holobiont is the integrated assemblage of the coral animal, its symbiotic algae, protists, fungi and a diverse consortium of *Bacteria* and *Archaea*. Corals are a model system for the study of symbiosis, the breakdown of which can result in disease and mortality. Little is known, however, about viruses that infect corals and their symbionts. Here we present metagenomic analyses of the viral communities associated with healthy and partially bleached specimens of the Caribbean reef-building coral *Diploria strigosa*. Surprisingly, herpes-like sequences accounted for 4–8% of the total sequences in each metagenome; this abundance of herpes-like sequences is unprecedented in other marine viral metagenomes. Viruses similar to those that infect algae and plants were also present in the coral viral assemblage. Among the phage identified, cyanophages were abundant in both healthy and bleaching corals and vibriophages were also present. Therefore, coral-associated viruses could potentially infect *all* components of the holobiont – coral, algal and microbial. Thus, we expect viruses to figure prominently in the preservation and breakdown of coral health.

## Introduction

Within a coral's skeleton, tissue and mucus, there exists a diverse assemblage of *Bacteria*, *Archaea*, algae, fungi and protists (Knowlton and Rohwer, 2003). Endosymbiotic algae, called zooxanthellae, and some *Bacteria* form relatively stable and species-specific associations with corals (Rohwer *et al*., 2002; Goulet, 2006). It has been hypothesized that the coral animal can adapt to differing ecological niches by ‘switching’ its algal and microbial associates. In the case of corals and zooxanthellae, this so-called adaptive bleaching may allow the coral animal to adjust to changing water temperatures (Buddemeier *et al*., 2004). Coral-associated *Bacteria* can serve as a food source for corals (Sorokin, 1973; Bak *et al*., 1998) and provide beneficial metabolic capabilities such as nitrogen fixation in at least one coral species (Lesser *et al*., 2004; 2007). It has been hypothesized that changes in microbe–coral associations will facilitate the survival of corals under future environmental changes (Reshef *et al*., 2006).

The least-studied constituents in the coral holobiont are the viruses. No cnidarian viruses have been isolated to sufficient purity to be identified genetically prior to this study, although viruses have been observed visually in association with corals and other cnidarians. An observation of virus-like particles (VLPs) in the zooxanthellae of anemones first implicated viruses in coral bleaching (Chapman, 1974; Wilson and Chapman, 2001). VLPs were later observed in the tissues of heat-shocked and control specimens of the scleractinian coral *Pavona danai* (Wilson *et al*., 2005) and in the tissue and zooxanthellae of three coral species and one species of zoanthid, all under thermal stress (Davy *et al*., 2006). The origin of these VLPs was not known. A recent study demonstrated that UV stress induced one type of latent virus in cultures of coral zooxanthellae (Lohr *et al*., 2007). In sum, observations of VLPs in corals have generally been made under the impression that their presence is an indicator of coral stress or disease (Wilson *et al*., 2005; Davy *et al*., 2006). However, given the abundance and diversity of coral-associated microbes, it is expected that these virus populations will consist of abundant and diverse bacteriophages in addition to viruses suspected to target eukaryotic cells, and that viruses will consistently be found in association with corals.

Viral genetic diversity is difficult to characterize because viruses share no single conserved sequence that can be used in a manner analogous to the sequencing of ribosomal RNA from cellular organisms (Rohwer and Edwards, 2002). Individual viruses contain extremely small amounts of DNA (Steward *et al*., 2000) and often use modified bases, making cloning difficult (Warren, 1980). Viruses also carry genes toxic to bacterial cloning hosts (Wang *et al*., 2000). Thus, in order to characterize an entire community of coral-associated viruses genetically, the viruses must be physically isolated from bacterial, archaeal, algal and host cells, as well as free DNA, *prior* to DNA extraction and cloning (Rohwer *et al*., 2001a). Here, a homogenization and centrifugation technique was developed to purify viruses from the tissues of healthy and partially bleached specimens of the Caribbean coral *Diploria strigosa*. Shotgun sequencing and metagenomic analyses were then used to determine the genetic content and diversity of these two viral communities. Our results show that coral-associated viruses are extraordinarily diverse and potentially infect all members of the coral holobiont.

## Results and discussion

### Isolation and sequencing of coral-associated viruses

**Fig. 1:**
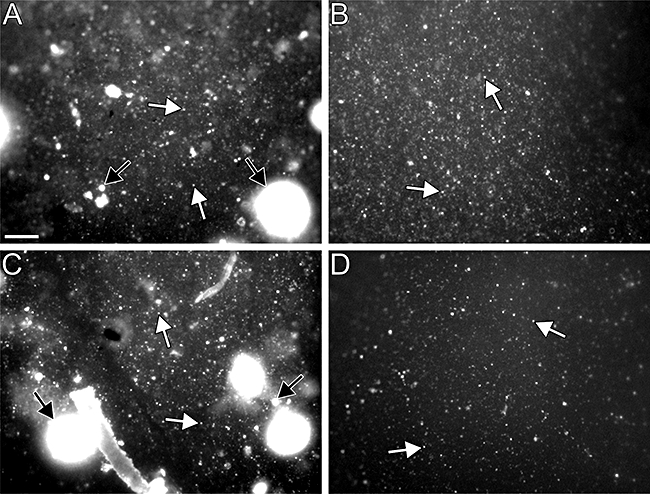
Epifluorescent micrographs of samples before and after viral particle isolation. DsH = *Diploria strigosa –* Healthy, DsB = *Diploria strigosa –* Bleaching. Samples were stained with SYBR Gold nucleic acid stain and visualized under epifluorescence at 1000×. A. and C. Whole coral blastate from (A) DsH and (C) DsB. Visible are abundant virus-like particles (VLPs, white arrows), as well as intact microbial cells and autofluorescent zooxanthellae (black arrows). B. and D. Purified viruses from (B) DsH and (D) DsB. VLPs are visible (white arrows) in addition to autofluorescence of coral GFP-like proteins. Scale bar represents approximately 5 μm and applies to all four panels.

Viral particles were physically isolated prior to DNA extraction and shotgun cloning. Fragments of healthy and partially bleached *D. strigosa* colonies were collected in triplicate. Bleaching corals were visually identified as those that had lost 40–60% of their normal pigmentation. Tissue was removed from coral skeletons using an airbrush. Tissue in each set of triplicate samples was pooled to create two metagenomic libraries, DsH and DsB. (‘Ds’ in the sample name represents the coral species, *D. strigosa*, while ‘H’ and ‘B’ indicate the corals' healthy or bleaching condition.) Pooling was not necessary to obtain sufficient viral material. However, we chose to pool samples to reduce the sample-to-sample variation associated with metagenomic data sets (Angly *et al*., 2006). Pooling samples also allowed us to examine a broader diversity of coral-associated viruses, though it concealed any variability between the individual corals collected. The raw coral tissue blastate was too viscous for processing, so power homogenization was used to liquefy it. Virus counts before and after homogenization showed no significant difference in VLP abundance (Table S1; *P* = 0.88). Centrifugation in cesium chloride density gradients was used to separate VLPs from cellular debris. Removal of microbial and eukaryotic cells was confirmed with epifluorescence microscopy (Fig. 1). To ensure that all microbial DNA was removed, the viral fraction from the cesium chloride gradient was treated with chloroform to lyse mitochondria. DNase I was then used to degrade the exposed DNA (Breitbart and Rohwer, 2005).

DNA was extracted from the isolated viruses and cloned using the Linker Amplified Shotgun Library (LASL) method. In total, 1580 sequences from the DsH library and 930 sequences from the DsB library were obtained. When compared with the GenBank non-redundant (NR) database using tblastx, 44% of DsH sequences and 59% of DsB sequences had significant hits to known sequences (*E*-value < 0.001; Table 1). When compared with the environmental sequence database (ENV), 60% of DsH and 77% of DsB sequences had significant hits (*E*-value < 0.001; Table 1). When NR and ENV hits were compiled, 35% of DsH and 18% of DsB sequences were entirely novel (i.e. they had no hits to either database). This percentage of novel sequences is lower than that observed in other viral metagenomes (Breitbart *et al*., 2002; 2003; 2004; Angly *et al*., 2006), but there still remains a large fraction of coral-associated viruses with no significant similarity to any known sequences.

**Table 1:** Summary of tblastx hits to GenBank NR and ENV databases.

	DsH hits		DsB hits	
	Number of hits	% of all sequences	Number of hits	% of all sequences
By database				
None	557	35.3	172	18.5
Environmental database only	324	20.2	208	22.4
GenBank + environmental database	626	39.6	508	54.6
GenBank only	73	4.6	42	4.5
Total sequences	1580		930	
By virus type				
Eukaryote-specific viruses	98	6.2	85	9.1
Herpesviruses	68	4.3	71	7.6
Phages	188	12	147	16
Cyanophages	55	3.5	64	6.9
Vibriophages	7	0.4	9	1.0
Total virus hits	286	18	232	25

Percentages are calculated from the total sequences in each metagenome. Virus hits are categorized based on the top hit to a virus sequence.

### Animal viruses associated with corals

Remarkably, 4.3% of all DsH sequences and 7.6% of all DsB sequences had significant hits to herpesviruses when compared with the NR database (Table 1). This represents 69% and 84% of the hits to eukaryote-specific viruses in the DsH and DsB libraries respectively. The most common herpesvirus hits were cercopithecine herpesvirus 2 sequences in the DsH library and cercopithecine herpesvirus 1 sequences in the DsB library (summarized in Table 2; listed in full in Table S2). These are both alphaherpesviruses, but significant hits to all subfamilies of the family *Herpesviridae* were observed.

**Table 2:** Summary of tblastx hits to GenBank NR database.

		Number of hits	
Family/subfamily	Virus name	DsH	DsB
*Alphaherpesvirinae*	Cercopithecine herpesvirus 2	11	7
*Alphaherpesvirinae*	Bovine herpesvirus 5	10	8
*Gammaherpesvirinae*	Saimiriine herpesvirus 2	8	7
*Alphaherpesvirinae*	Cercopithecine herpesvirus 1	6	13
*Alphaherpesvirinae*	Suid herpesvirus 1	5	7
*Alphaherpesvirinae*	Ateline herpesvirus 3	4	2
*Alphaherpesvirinae*	Human herpesvirus 1	4	0
*Alphaherpesvirinae*	Bovine herpesvirus 1	3	5
*Alphaherpesvirinae*	Human herpesvirus 2	3	4
*Gammaherpesvirinae*	Human herpesvirus 8	3	7
*Phycodnaviridae*	*Emiliania huxleyi* virus 86 isolate EhV86	4	4
*Phycodnaviridae*	*Ectocarpus siliculosus* virus	1	0
*Phycodnaviridae*	*Paramecium bursaria* Chlorella virus 1	3	3
*Phycodnaviridae*	*Chlorella* virus	1	0
*Podoviridae*	Cyanophage P-SSP7	18	41
*Podoviridae*	Cyanophage P60	15	7
*Myoviridae*	Cyanophage P-SSM2	13	13
*Myoviridae*	Cyanophage P-SSM4	4	2
*Podoviridae*	Vibriophage VP2	4	1
*Podoviridae*	Vibriophage VP5	1	3
*Myoviridae*	*Vibrio harveyi* bacteriophage VHML	0	3

Listed are the most commonly hit viruses in four categories: herpesviruses, algae viruses, cyanophages and vibriophages (*E*-value < 0.001). Herpesviruses are classified by subfamily, other viruses by family. Full tblastx hits are presented in Table S6.

**Fig. 2:**
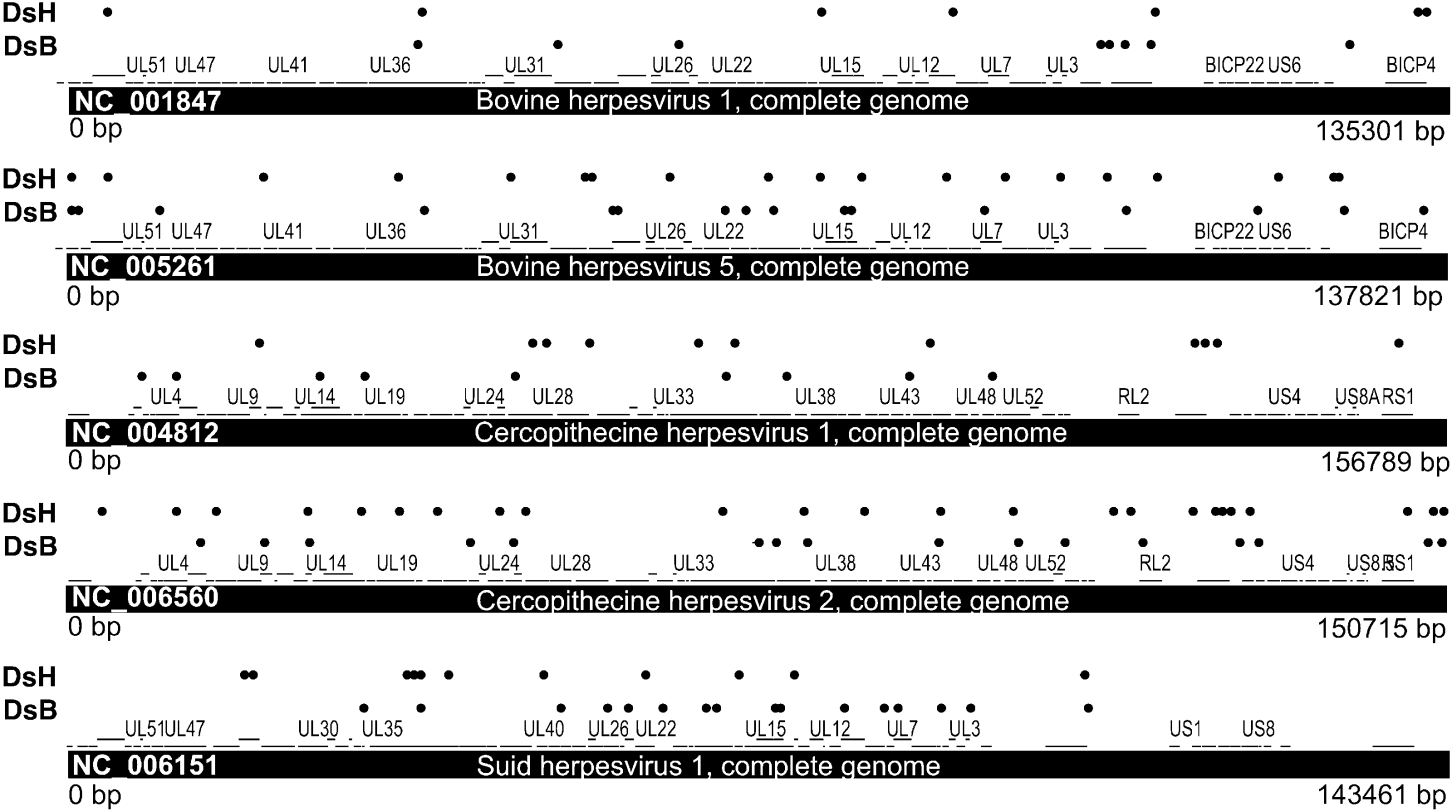
Alignment of coral virus sequences to alphaherpesvirus genomes. Each dot represents the location of an individual metagenome sequence based on its best tblastx hit (*E*-value < 0.001) to the virus genome. Shown are the five alphaherpesviruses with the most hits.

Two features of the tblastx hits indicate that the coral viral community did not contain known herpesviruses, but rather ‘herpes-like’ viruses. First, sequence similarity was rarely above 70% amino acid identity. Second, many sequences hit simple repeats in complete herpesvirus genomes. These repeats are characteristic of herpesviruses (McGeoch *et al*., 2006), but not diagnostic given the level of sequence divergence. To better visualize these herpes-like sequences, we used the location of the best tblastx hit for each sequence to plot sequence hit along the most commonly identified herpesvirus genomes (Fig. 2). These hits were distributed fairly evenly across the target genomes, indicating that there are many regions of similarity. This supports the identification of these viruses as ‘herpes-like’.

To better characterize sequences functionally, DsH and DsB were compared with a database of complete, annotated genomes from eukaryote-specific viruses using blastx (*E*-value < 0.001; accession numbers of genomes included in the database are listed in Appendix S1). Eight sequences from each metagenome had a significant hit to a herpesvirus gene (Table S3). This included a total of four hits to genes involved in nucleotide metabolism (e.g. ribonucleoside-phosphate reductase), four hits to predicted or known glycoprotein genes, and four hits to genes for latency-associated nuclear antigens. The small number of hits demonstrates the high degree of sequence novelty in the coral-associated viral communities and further supports the notion that the ‘herpes-like’ viruses associated with corals are highly divergent from any previously studied viruses. Nevertheless, the tblastx and blastx results together show that a subset of coral-associated viruses is more similar to herpesviruses than to anything else known.

Herpes-like sequences did not comprise a large percentage of hits in previously published viral metagenomes from nearshore seawater, global ocean seawater, marine sediment or human feces (Breitbart *et al*., 2002; 2003; 2004; Angly *et al*., 2006). The occurrence of these sequences at such high abundance is therefore novel to corals. This should expand the scope of research on coral disease, but more work is needed before the potential pathogenicity of these viruses is known.

### Algae and plant viruses associated with corals

Hits to viruses that infect algae and plants (family *Phycodnaviridae*) were also observed in the tblastx comparison to the NR database (summarized in Table 2, listed in full in Table S2). The DsH library contained one hit to *Ectocarpus siliculosus* virus, a virus of brown algae. The DsH and DsB libraries included three and four hits, respectively, to chlorella viruses, which infect green algae. Each library also contained four hits to *Emiliania huxleyi* virus 86, a virus of coccolithophores. The presence of phycodnavirus genes in the ‘bleaching’ DsB sequences is consistent with the fact that this metagenome was created from corals that had only partially bleached when they were collected. This tissue therefore still contained large numbers of symbiotic algae, which could serve as targets for phycodnaviruses.

When compared with the database of virus genomes using blastx, 30 DsH sequences and 22 DsB sequences had significant hits to phycodnavirus genes (Table S4; *E*-value < 0.001). Nearly all of these genes coded for hypothetical or putative proteins. Most functional predictions, when available, involved nucleotide metabolism (e.g. thymidylate synthase and ribonucleoside-triphosphate reductase). Although the phycodnavirus hits comprised fewer than 10% of hits to eukaryote-specific viruses in both tblastx and blastx searches, their presence is notable because some phycodnaviruses are known to infect the symbiotic microalgae of hydra (Van Etten *et al*., 1982). This collection of hits suggests that a subset of coral-associated viruses may target algal cells in the coral holobiont. Thus, the potential remains for coral viruses to contribute to coral bleaching by directly infecting zooxanthellae, as researchers have previously posited (Wilson *et al*., 2005).

### Coral-associated phages

When compared with the NR database with tblastx, 29% of the phage hits in DsH and 44% of the phage hits in DsB were to cyanophage sequences (summarized in Table 2; full list in Table S2). These phages represent a guild that is known to infect cyanobacteria rather than a taxonomic group of phages. For example, cyanophages P-SSP7 and P60 are podoviruses, but cyanophages P-SSM2 and P-SSM4 are myoviruses. DsH and DsB both contained sequences with high similarity to all four of these cyanophages.

To further characterize the phage hits, DsH and DsB were compared with a database containing only phage genes (the Phage Sequence Databank, http://phage.sdsu.edu/phage) using blastx (*E*-value < 0.001). A total of 173 DsH sequences and 153 DsB sequences had significant hits to cyanophage genes. These hits represented nearly all functional categories of cyanophage genes, including tail and capsid components, nucleotide metabolism, DNA replication, DNA repair and protein translation (Table S5). Interestingly, there were extremely strong hits in both libraries to the core photosystem II reaction centre protein encoded by the *psbA* gene. To ensure that these photosystem hits were not derived from coral zooxanthellae symbionts (*Symbiodinium* spp.), which also have *psbA* genes, each sequence was compared with the GenBank NR database using blastn (*E*-value < 0.001). The NR database contains *psbA* genes from four different *Symbiondinium* clades; however, there were no significant hits to these sequences in any of the comparisons. The core photosystem II reaction centre proteins encoded by the *psbA* gene were recently shown to be present and functional in cyanophage genomes (Sullivan *et al*., 2006). Their presence here lends supports to the identification of these coral-associated viruses as cyanophages.

The discovery of cyanophages in the coral viral assemblage is not unexpected. Cyanobacteria were previously identified in one of four 16S rDNA clone libraries from *D. strigosa*-associated *Bacteria* (Rohwer *et al*., 2002) and as endosymbionts in the Caribbean coral *Montastraea cavernosa* (Lesser *et al*., 2004). The cyanobacteria in *M. cavernosa* belong to the Order *Chroococcales*. This Order also contains *Prochlorococcus* spp. and *Synechococcus* spp., which are the targets of the cyanophages identified in the DsH and DsB libraries.

It has been proposed that cyanobacterial symbionts of corals perform nitrogen fixation within either the coral skeleton or tissue (Shashar *et al*., 1994; Lesser *et al*., 2004). Nitrogen fixation within the tissue has now been demonstrated in *M. cavernosa* (Lesser *et al*., 2007). Zooxanthellae are typically thought to be nitrogen limited, but the authors of this study showed that zooxanthellae could acquire fixed nitrogen directly from endosymbiotic cyanobacteria. Thus, the infection of cyanobacteria by cyanophages could determine the ability of zooxanthellae to acquire fixed nitrogen within the coral holobiont.

Vibriophages made up 3.7% and 6.0% of DsH and DsB phage hits, respectively, to the NR database (summary in Table 2, full list in Table S2). When compared with the Phage Sequence Databank using blastx, DsH and DsB contained 67 and 80 hits, respectively, to vibriophage genes, including those involved in tail construction, protein translation, nucleotide metabolism, and DNA packaging, replication and repair (Table S6).

A subset of *Vibrio* spp. known to cause coral bleaching and disease has become the basis for model systems used to examine the interactive effects of microbes and temperature on coral physiology (Kushmaro *et al*., 2001; Ben-Haim *et al*., 2003). Recently, phage therapy with cultured vibriophages was shown to prevent tissue necrosis and bleaching in *Pocillopora damicornis* specimens experimentally infected with the bacterium *Vibrio coralliilyticus* (Efrony *et al*. 2007). Vibriophages in the coral holobiont could similarly infect bacterial pathogens and therefore benefit the coral; however, their presence could be detrimental if they disrupt the coral holobiont (e.g. by releasing toxins or clearing space for more virulent strains). Further investigations should be conducted to determine the degree of influence that vibriophages have on their target populations in the complex environment of the coral holobiont.

The abundance of cyanophages and vibriophages in these sequence libraries should not be taken to represent precise phage abundances in nature. These two groups are well studied and well represented in GenBank. As other microbial symbionts of corals are identified and their phages are studied, the relative abundances of cyanophages and vibriophages identified in coral viral metagenomes will decrease. The functional capacities of these two groups, however, will remain an important focus of attention.

### Statistical comparison of phage communities

UniFrac (Lozupone *et al*., 2006), a metric of the unique phylogenetic distance between communities (Lozupone and Knight, 2005), was used to compare the phage populations in our coral virus metagenomes to each other and to two previously obtained phage sequence libraries: Reef (from water collected at four coral reefs) and Ocean (a pooling of sequences from four oceanic provinces; Angly *et al*., 2006). There was no significant difference between the DsB and DsH samples in this analysis (*P*= 0.94). A clustering of environments based on the UniFrac metric showed that these communities were more similar to each other than to the Ocean or Reef communities, with 100% jackknife support for all nodes. Because this method compares metagenome sequences to a phylogeny of known phages, it cannot reveal differences in phages that are undescribed. Because we chose to pool tissue samples prior to DNA extraction, this method also cannot reveal variability between individuals. In the context of known phage families, the coral phage communities do not have significant differences from each other, however, they do cluster together when compared with other marine phage communities, which suggests that the coral holobiont is a distinct environment from that of the surrounding seawater. This falls in line with previous studies demonstrating that coral bacterial communities differ from those in the surrounding seawater (Rohwer *et al*., 2001b; Frias-Lopez *et al*., 2002; Ritchie, 2006).

### Viral community structure

To characterize the sequence diversity of coral-associated viral communities as a whole, metagenome sequences were assembled into groups of contiguous sequences (contigs). The number of sequences in each contig was tallied and the frequencies of contigs of each size were used to predict characteristics of the viral communities (Breitbart *et al*., 2002; Angly *et al*., 2005) using PHACCS (Angly *et al*., 2005). The DsH metagenome contained 1523 singletons, 22 contigs of two sequences, three contigs of three sequences and one contig of four sequences (thus, the contig spectrum was [1523, 22, 3, 1]; Table S7). In the DsB metagenome, contig construction yielded only singletons and two-sequence contigs, which are not considered sufficient for accurate modelling. Based on its contig spectrum, the DsH community was predicted to have a total of 28 600 viral types and a Shannon–Weiner index of 8.96. The most dominant genotype was predicted to comprise 2.6% of the total viral community. This is extraordinary diversity, comparable to that of viral communities in soil (Fierer *et al*., 2007) and more diverse than viral communities in seawater (Breitbart *et al*., 2002).

### Implications for the coral holobiont

Interest in coral microbiology has surged with the recognition that coral-associated microbes may serve as pathogens or as mutualists that perform nitrogen fixation, vitamin and nutrient scavenging, antimicrobial production or space filling. The relative importance of each potential role has yet to be elucidated; however, bacteriophages should be expected to affect these microbial communities and their functionality. Phages are responsible for 50% of bacterial death in the ocean (Wilcox and Fuhrman, 1994) but may be responsible for a much higher percentage in the coral holobiont if the mobility of protist predators is restricted by the coral's tissue and mucus. Thus, the differential infection of bacterial groups by phages might serve as a ‘top-down’ control on the diversity of the coral-associated microbial consortium and its ability to fill the various roles described above.

The coral animal itself is also expected to regulate its microbial communities. It was demonstrated that sterilized coral mucus acts as a selective agent, promoting the growth of non-pathogenic and antibiotic-producing bacterial strains over known pathogens; this selectivity was not observed when mucus was collected during a bleaching event (Ritchie, 2006). Changes in coral mucus chemistry over long (evolutionary) or short (ecological) timescales can therefore provide ‘bottom-up’ control over a community of microbial associates. The relative extent to which a coral or its phage population is able to exert such control on a microbial community is not yet known, but this study shows that further research is needed to understand the role of phages in structuring these communities.

Microbial abundances in coral tissues are approximately 10^7^ per cm^2^ (Wegley *et al.*, 2004). In most environments, there are 10 VLPs for every microbial cell, the majority of which are phage. If this held true for the coral holobiont microenvironment, we would expect corals to have 10^8^ VLPs per cm^2^. Considering the large number of hits to eukaryote- and algae-specific viruses in our metagenomic libraries, the typical viral abundance on corals might be much greater. While the degree of natural variability in coral virus abundance remains to be determined, we propose that observations of extraordinary numbers of viruses in apparently healthy corals may represent a diversity of functions rather than a severity of infection.

Studies of coral-associated viruses often describe these communities as a latent pathogen reservoir, susceptible to induction by environmental stressors. The presence of eukaryote-specific viruses is demonstrated by the sequence data presented here, and it is indeed likely that some coral pathologies are caused by viral vectors. However, it appears that corals are chronically infected by thousands of viral strains. The DNA used to create our metagenomes was extracted from viral particles, thus these sequence libraries represent not latent viral sequence embedded within eukaryote genomes but viral particles present in the coral tissue at the time of collection. The presence alone of viruses is therefore not indicative of disease.

Furthermore, the observed genetic diversity of viruses in the coral holobiont casts the very nature of coral symbiosis in a new light. Lesser and colleagues (2007) showed that while coral-associated cyanobacteria could provide fixed nitrogen to zooxanthellae, zooxanthellae did not appear to depend on this source of nitrogen. Therefore, the mechanism maintaining coral–zooxanthellae symbiosis was not fully understood. Villarreal (2007) has suggested that viruses can serve as a stabilizing force for symbioses by establishing addiction systems within a host. For example, zooxanthellae infected with a latent virus are resistant to lysis by VLPs (Wilson and Chapman, 2001). Phages may also stabilize symbioses. For example, a phage specific to *Hamiltonella defensa*, a bacterial symbiont of the pea aphid, produces a toxin that appears to protect host aphids from eukaryotic parasites (Moran *et al*., 2005). The diversity of constituents in the coral holobiont elevates the potential for these types of viral functions to exist therein. In fact, the stability of the holobiont itself may ultimately depend on the action of viruses. The study of viruses within the coral holobiont will shed new light on the basic biology of symbiosis, but it will also be particularly important as corals face ever-increasing threats to their health and habitats.

Here we have described the complexity of an under-studied facet of the coral holobiont. Herpes-like viruses occur in both healthy and bleaching corals. This should be a focus for future research on coral holobiont complexity, symbiosis and immunology. The largest identified functional group of coral-associated viruses, cyanophages, may affect the population structure of symbiotic cyanobacteria and endolitic algae, while vibriophages present in coral tissue may affect the pathogenesis of coral-associated *Vibrio* spp. While these are important structuring forces for the coral holobiont, the prediction that up to 28 600 viral types occur in a healthy coral's viral community indicates that there are myriad functions and interactions still unidentified in this viral assemblage. When compared in the framework of a phage phylogenetic tree, coral-associated phage communities from bleaching and healthy corals are not significantly different from each other, but the coral holobiont as a phage environment is distinct from that of coral reef and oceanic waters. Thus, it appears that a diverse community of viruses continuously occupies coral tissues. With the potential to target animal, algal and microbial cells, viruses are likely to be crucial in maintaining the overall function of the coral holobiont.

## Experimental procedures

### Sample collection and preservation

Coral fragments were collected on 15 March 2005 from the fringing reef at Mount Irvine Bay (GPS coordinates: 11°11′45′′N; 60°47′54′′W) in Buccoo, Tobago. Corals were collected close to shore at a depth of 3.7 m using a hammer and chisel on SCUBA. Bleaching colonies were identified visually as those that had lost 40–60% of their normal pigmentation and lacked any apparent disease or tissue loss. Individual fragments (approximately 5 cm diameter) were placed in plastic bags and transported to the laboratory. For each sample, coral fragments were collected in triplicate from different colonies and blastate was pooled after airbrushing (see below).

Within 1 h of collection, coral tissue was processed and preserved. Corals were rinsed with filtered, autoclaved sea water (FASW) to dislodge any loosely associated microbes, viruses and sediments. Coral tissue, mucus and microbes were then removed from the coral skeleton with an airbrush (40 psi/2.8 bar) and filtered, autoclaved seawater. Coral blastate was collected in plastic bags and transferred to 50 ml conical tubes. Chloroform was added (approximately 5 ml per 40 ml blastate) to kill all cells. Samples were stored at 4°C.

### Microscopy of coral virus-like particles

Samples were diluted in FASW, fixed with paraformaldehyde (final concentration 4% v/v), and filtered onto 0.02 μm glass filters (Anodisc; Whatman Inc., Clifton, NJ, USA) using vacuum suction (8 psi/0.6 bar). Filters were incubated with 1× SYBR Gold fluorescent nucleic acid stain (10 min; Invitrogen, Carlsbad, CA, USA) and mounted onto glass slides. Filters were viewed at 1000× magnification using epifluorescence microscopy with a FITC filter. Images were captured and viral particles were quantified with Image Pro Plus software (Media Cybernetics, Silver Spring, MD).

### Isolation of viral DNA from coral blastates

Coral blastates were liquefied using a handheld homogenizer (PowerGen, 5000 r.p.m., Fisher Scientific, Pittsburg, PA) and centrifuged at 3000 r.p.m. for 15 min to remove sediments and large debris. Supernatant was transferred to sterile glass Corex tubes and centrifuged at 10 000 r.p.m. for 15 min to pellet the majority of microbial cells (SS-24 rotor, 12 000 *g*). Cleared coral blastates were loaded onto cesium chloride (CsCl) step gradients (1.35, 1.5, 1.7 g ml^−1^ CsCl in FASW) in polycarbonate tubes and spun in an ultracentrifuge at 22 000 r.p.m. for 2 h at 4°C (SW-41 rotor, 600 000 *g*). The viral fraction, at the junction of 1.35 and 1.5 g ml^−1^ density fractions, was collected using a sterile syringe as described by Steward and colleagues (2000).

To eliminate any remaining microbes, mitochondria, and free DNA, 150 μl of chloroform was added to each isolated phage fraction and samples were agitated. To digest resulting free microbial DNA, samples were incubated with DNase I (1 unit per 100 μl sample) at 37°C for 2 h. This method was previously used to isolate purified viruses from human blood and fecal samples (Breitbart and Rohwer, 2005; Zhang *et al*., 2006). Aliquots were prepared for microscopy after each isolation step to ensure retention of viral particles.

DNase I was inactivated by the addition of EDTA at the beginning of the viral lysis procedure (final concentration 0.025 M). Viral capsids were disrupted with a formamide extraction. DNA was purified with isopropanol precipitation and a CTAB (hexadecyltrimethylammonium bromide) extraction (Sambrook *et al*., 1989).

### Shotgun library construction

Viral DNA was amplified and cloned by constructing linker-amplified shotgun libraries (LASLs, Lucigen Corp, Middleton, WI). DNA was mechanically sheared using a HydroShear (GenMachine, San Carlos, CA) and resultant fragments were ligated to common sequence linkers. Primers specific to the linkers were used to randomly amplify the entire DNA population with Vent DNA polymerase. Amplified DNA was cloned into pSMART vectors and electroporated into MC12 cells. This method has been shown to amplify sequences randomly from total environmental DNA (Rohwer *et al*., 2001a).

### Sequence processing

Plasmids containing viral DNA inserts were sequenced on capillary sequencers (Agencourt Biosciences, Beverly, MA and SymBio Corp, Menlo Park, CA). PHRED was used to call bases and to trim vector and adapter sequences (Ewing and Green, 1998; Ewing *et al*., 1998). Base quality was scored using PHRAP (Green, 1994). Bases with PHRAP scores < 20 were masked with an N. Sequences were trimmed further to remove ends containing fewer than 50 unambiguous bases by using FastGroupII (Yu *et al*., 2006). Trimmed sequences shorter than 100 bp were eliminated from analyses.

Sequences were assembled into contigs using TIGR Assembler (Pop and Kosack, 2004). Chromatograms from all contigs were visually inspected for the occurrence of identical sequences, which result from sequencing a bacterial clone that has grown in two neighbouring culture wells. Duplicate sequences of this kind were removed from analyses. The DsH library yielded a total of 1580 high-quality sequences; the DsB library yielded 930. From sequence assemblies, contig spectra were determined and were used to model viral community structure with the PHACCS (PHAge Communities from Contig Spectrum) online tool (Angly *et al*., 2005).

### Sequence comparison to GenBank and specialized databases

Sequences were compared with the GenBank NR and NCBI ENV databases using tblastx (Altschul *et al*., 1990; 1997). Significant hits were defined as matches with *E*-values less than 0.001. For summary statistics, sequences were sorted based on hits to each of the two databases. For analysis of viral types, sequences were categorized based on the top viral hit and further sorted based on viral host (microbe or eukaryote).

Sequences were then compared with two smaller databases using blastx (*E*-value < 0.001). First, all sequences were compared with a database of complete genomes from eukaryote-specific viruses. These genome sequences are curated by RefSeq and were downloaded from the NCBI Viral Genomes Resource on 20 August 2007 (http://www.ncbi.nlm.nih.gov/genomes/VIRUSES/10239.html; complete list of accession numbers provided in Appendix S1). The resulting database of 1974 viral genomes contained a total of 28 456 annotated proteins. Second, sequences were compared with the Phage Sequence Databank (http://phage.sdsu.edu/phage; version released 5 December 2006), which contains 510 complete phage genomes as well as manually curated phage and prophage sequences. A list of blastx hits and a list of accession numbers used in the eukaryote-specific virus database are available from the authors. To visualize the distribution of sequence hits across viral genomes, sequences were compared with the same two databases using tblastx (*E*-value cut-off = 0.001) and the best hit was used to plot each sequence onto a viral genome.

### UniFrac test of community similarity

Sequence libraries were compared with a database of all complete phage genomes with blastn (significance cut-off of *E*-value< 0.01). Significant hits were transposed onto a previously constructed multiprotein phylogenetic tree of the phage genomes (Rohwer and Edwards, 2002). The tree topology and the distribution of hits along this tree were uploaded to the UniFrac online computational platform (Lozupone and Knight, 2005; Lozupone *et al*., 2006). The Cluster Environments analysis was used to group environments based on the similarity of the viral lineages contained within each (100 jackknife permutations, Use abundance weights = True). The UniFrac significance test was used to compare pairs of viral communities (100 permutations, Use abundance weights = True).

## Supplementary material

The following supplementary material is available for this article online:

Appendix S1. Accession numbers of sequences included in database of eukaryote-specific virus genomes. Genome sequences are curated by RefSeq. All complete genomes from eukaryote-specific viruses were downloaded from the NCBI Viral Genomes Resource (http://www.ncbi.nlm.nih.gov/genomes/VIRUSES/10239.html) on 20 August 2007.

**Table S1.** Viral particle abundance in raw and homogenized DsH tissue blastate. Viral particles were counted in homogenized and un-homogenized coral blastate using epifluorescence microscopy to determine whether mechanical homogenization resulted in shearing and loss of viral particles.

**Table S2.** tblastx hits to virus sequences in GenBank. Sequence libraries DsH and DsB were compared with the GenBank NR database using an *E*-value cut-off of 0.001. The top virus sequence hit for each metagenome sequence was used to calculate totals. Percentages are calculated from the total hits in each of two categories: sequences from eukaryote-specific viruses and sequences from phages. Hits to herpesvirus sequences are sorted according to their subfamily within the family *Herpesviridae*; all other virus hits are sorted according to virus family, if such classification exists. Hits to phage sequences are categorized by family according to the Phage Proteomic Tree and further sorted by phage morphology. ND = no classification data available.

**Table S3.** blastx hits to herpesvirus genes. Sequences were compared with a database of all complete genomes from eukaryote-specific viruses. *E*-value cut-off = 0.001. Only the top hit for each metagenome sequence is listed.

**Table S4.** blastx hits to genes from algae viruses (family *Phycodnaviridae*). Sequences were compared with a database of all complete, annotated genomes from eukaryotespecific viruses. *E*-value cut-off = 0.001. Only the top hit for each metagenome sequence is listed.

**Table S5.** *blastx* hits to cyanophage genes in the Phage Sequence Databank (http://phage.sdsu.edu/phage; *E*-value < 0.001). All metagenome sequences with best hits to cyanophage genes were compiled. Listed are the 15 genes with the lowest *E*-value in each search and any gene with four or more hits. blastx statistics are listed for the best hit to each gene. Hits to hypothetical and unknown targets are not shown.

**Table S6.** blastx hits to vibriophage genes. Sequence libraries were compared with the Phage Sequence Databank using an *E*-value cut-off of 0.001. All metagenome sequences with best hits to vibriophage genes were compiled. Listed are the 10 genes with the lowest *E*-value in each search, as well as any gene with three or more hits. blastx statistics are listed for the best hit to each gene. Hits to hypothetical and unknown targets are not shown.

Table S7. Diversity and structure of the viral community in healthy *Diploria strigosa* tissues. Contig spectrum was tallied from metagenome sequence overlaps and used to predict aspects of viral community structure and diversity using the PHACCS online tool.

This material is available as part of the online article from http://www.blackwell-synergy.com
