# Supplementary material for: Viral communities associated with healthy and bleaching corals
Source: Environ Microbiol. 2008 Sep;10(9):2277–86. doi: 10.1111/j.1462-2920.2008.01652.x (PMC2702503; doi:10.1111/j.1462-2920.2008.01652.x)
Supplement: Appendix S1. — Accession numbers of sequences included in database of eukaryote-specific virus genomes. Genome sequences are curated by RefSeq. All complete genomes from eukaryote-specific viruses were downloaded from the NCBI Viral Genomes Resource (http://www.ncbi.nlm.nih.gov/genomes/VIRUSES/10239.html) on 20 August 2007. [file emi0010-2277-SD1.doc]

Appendix S1. Accession numbers of sequences included in database of Eukaryote-specific virus genomes. Genome sequences are curated by RefSeq. All complete genomes from Eukaryote-specific viruses were downloaded from the NCBI Viral Genomes Resource (http://www.ncbi.nlm.nih.gov/genomes/VIRUSES/10239.html) on August 20, 2007.

NC_007370 NC_004273 NC_006001 NC_002501 NC_004642 NC_004219 NC_002561 NC_003847 NC_006458 NC_001946 NC_004658 NC_007213 NC_003703 NC_003501 NC_007467 NC_002188 NC_003891 NC_004067 NC_002687 NC_006312 NC_003562 NC_003740 NC_001918 NC_006499 NC_002067 NC_001928 NC_004220 NC_003398 NC_002691 NC_005030 NC_006446 NC_007638 NC_006308 NC_001441 NC_007013 NC_004264 NC_005850 NC_007671 NC_004645 NC_005359 NC_002018 NC_004292 NC_003443 NC_002558 NC_004020 NC_004553 NC_002038 NC_005842 NC_005290 NC_002690 NC_007367 NC_006005 NC_004185 NC_001547 NC_002021 NC_003467 NC_007915 NC_007656 NC_005897 NC_006506 NC_003791 NC_005894 NC_004637 NC_006146 NC_000898 NC_005147 NC_001672 NC_002531 NC_001866 NC_003835 NC_003874 NC_006004 NC_000870 NC_003775 NC_005330 NC_006950 NC_006319 NC_003651 NC_003008 NC_003867 NC_005289 NC_003753 NC_002644 NC_003503 NC_003976 NC_002028 NC_003519 NC_006944 NC_003378 NC_001474 NC_004638 NC_006275 NC_007525 NC_002803 NC_007803 NC_006631 NC_003347 NC_006658 NC_011451 NC_004211 NC_007469 NC_003794 NC_003664 NC_005979 NC_003011 NC_002207 NC_003754 NC_002815 NC_004009 NC_005853 NC_001885 NC_003661 NC_007357 NC_007551 NC_003461 NC_001753 NC_004268 NC_003865 NC_002657 NC_001846 NC_003873 NC_005216 NC_003546 NC_003616 NC_004515 NC_002026 NC_003660 NC_003013 NC_007903 NC_002520 NC_004221 NC_007374 NC_002036 NC_002634 NC_007918 NC_001983 NC_001364 NC_002797 NC_001461 NC_004018 NC_004640 NC_007468 NC_003084 NC_006643 NC_004216 NC_005895 NC_007465 NC_001652 NC_003828 NC_004099 NC_004052 NC_001648 NC_007744 NC_003738 NC_001265 NC_001795 NC_007000 NC_005819 NC_003625 NC_006554 NC_001494 NC_003837 NC_011452 NC_006453 NC_003796 NC_004100 NC_004188 NC_001524 NC_005996 NC_004422 NC_006563 NC_003878 NC_004284 NC_003401 NC_001806 NC_003391 NC_001483 NC_002209 NC_004810 NC_003632 NC_007151 NC_000883 NC_002728 NC_005817 NC_002557 NC_003648 NC_004355 NC_003451 NC_006553 NC_006444 NC_001724 NC_001819 NC_002361 NC_001403 NC_007212 NC_001455 NC_002198 NC_003746 NC_004706 NC_001366 NC_001831 NC_001593 NC_007667 NC_002048 NC_004063 NC_002470 NC_007537 NC_003851 NC_003770 NC_003757 NC_002206 NC_004650 NC_004288 NC_006957 NC_004050 NC_007532 NC_006023 NC_007346 NC_004581 NC_003403 NC_006577 NC_007067 NC_004213 NC_003020 NC_001746 NC_007218 NC_007044 NC_004676 NC_003678 NC_007566 NC_002548 NC_003570 NC_004673 NC_001499 NC_002588 NC_006459 NC_003508 NC_003731 NC_003219 NC_007016 NC_001531 NC_003840 NC_004096 NC_001798 NC_003705 NC_003516 NC_007904 NC_001782 NC_004218 NC_001437 NC_007383 NC_001344 NC_005173 NC_001611 NC_001886 NC_005050 NC_004723 NC_003436 NC_001482 NC_003787 NC_004626 NC_004613 NC_007371 NC_005883 NC_004732 NC_004256 NC_004255 NC_006566 NC_003767 NC_003877 NC_002990 NC_003766 NC_007659 NC_001618 NC_004187 NC_003492 NC_001944 NC_006265 NC_004186 NC_001278 NC_007742 NC_003480 NC_004365 NC_002357 NC_003369 NC_006019 NC_003215 NC_001678 NC_006271 NC_006266 NC_005096 NC_004182 NC_007543 NC_005174 NC_003779 NC_005166 NC_003018 NC_002046 NC_006649 NC_001868 NC_004751 NC_005302 NC_004583 NC_003663 NC_001634 NC_007561 NC_006507 NC_007415 NC_003809 NC_001543 NC_003499 NC_004044 NC_001662 NC_002042 NC_006435 NC_007032 NC_007359 NC_001828 NC_006316 NC_004500 NC_004995 NC_003743 NC_002044 NC_004733 NC_001801 NC_003476 NC_005775 NC_004194 NC_006961 NC_007034 NC_001514 NC_004279 NC_003199 NC_000852 NC_001607 NC_004184 NC_004101 NC_001352 NC_004006 NC_004199 NC_005028 NC_003842 NC_004905 NC_001491 NC_002232 NC_004148 NC_003689 NC_007216 NC_004204 NC_005134 NC_006638 NC_007723 NC_005906 NC_001477 NC_005898 NC_007747 NC_006496 NC_006016 NC_003556 NC_001449 NC_006269 NC_005038 NC_000869 NC_007767 NC_004156 NC_003554 NC_006995 NC_001546 NC_002556 NC_002729 NC_004451 NC_007017 NC_001914 NC_002043 NC_000874 NC_003709 NC_007541 NC_005068 NC_007914 NC_003631 NC_004049 NC_003656 NC_002617 NC_006966 NC_007572 NC_001973 NC_005997 NC_003765 NC_003830 NC_004830 NC_004346 NC_003781 NC_003799 NC_004453 NC_004731 NC_001408 NC_003056 NC_002052 NC_003477 NC_005158 NC_001809 NC_006443 NC_007735 NC_001731 NC_004300 NC_001642 NC_006259 NC_001768 NC_003568 NC_007571 NC_001710 NC_001489 NC_007028 NC_007584 NC_003414 NC_001690 NC_003748 NC_001683 NC_001693 NC_001369 NC_005986 NC_002164 NC_004262 NC_006267 NC_007368 NC_006651 NC_007605 NC_002795 NC_007522 NC_002509 NC_004123 NC_001409 NC_002534 NC_003541 NC_007039 NC_007642 NC_004908 NC_002350 NC_004662 NC_002200 NC_006633 NC_007640 NC_006437 NC_004274 NC_003771 NC_002633 NC_007409 NC_003745 NC_001867 NC_007160 NC_004363 NC_001463 NC_004198 NC_002199 NC_002068 NC_005860 NC_003601 NC_006650 NC_000936 NC_003807 NC_007380 NC_001552 NC_004265 NC_005237 NC_001587 NC_007726 NC_007031 NC_004036 NC_001605 NC_001544 NC_007661 NC_004271 NC_002064 NC_006941 NC_004110 NC_002549 NC_007523 NC_005051 NC_003031 NC_001916 NC_002032 NC_003971 NC_005285 NC_003630 NC_004017 NC_005159 NC_003741 NC_002801 NC_006261 NC_005889 NC_007923 NC_003102 NC_007559 NC_001829 NC_003059 NC_006564 NC_004641 NC_004012 NC_003735 NC_004800 NC_006875 NC_002701 NC_007536 NC_006632 NC_001874 NC_003876 NC_004162 NC_006500 NC_004618 NC_001964 NC_005846 NC_002330 NC_001897 NC_006455 NC_004911 NC_007737 NC_003797 NC_004824 NC_001545 NC_005965 NC_001598 NC_001632 NC_001557 NC_003838 NC_003861 NC_006639 NC_004289 NC_003803 NC_003621 NC_005137 NC_006646 NC_005156 NC_000858 NC_004048 NC_005060 NC_004579 NC_003266 NC_007377 NC_004280 NC_006934 NC_003708 NC_001780 NC_007751 NC_003113 NC_006635 NC_002066 NC_006457 NC_007549 NC_003691 NC_007463 NC_001871 NC_007455 NC_002324 NC_006310 NC_002641 NC_003473 NC_004362 NC_003723 NC_004690 NC_001503 NC_004102 NC_004635 NC_003782 NC_003676 NC_004107 NC_005171 NC_006555 NC_003634 NC_004423 NC_006568 NC_001407 NC_002551 NC_004666 NC_003505 NC_005896 NC_004005 NC_007553 NC_001917 NC_004750 NC_007658 NC_006436 NC_003557 NC_003680 NC_007473 NC_005343 NC_003005 NC_004014 NC_003987 NC_003822 NC_007564 NC_005888 NC_007381 NC_003772 NC_003690 NC_003357 NC_005881 NC_006942 NC_001694 NC_005167 NC_006647 NC_004168 NC_007647 NC_006434 NC_005790 NC_005994 NC_004201 NC_003647 NC_004660 NC_002566 NC_003833 NC_007369 NC_003760 NC_007646 NC_006056 NC_006009 NC_003012 NC_007290 NC_001671 NC_001948 NC_001927 NC_003649 NC_006052 NC_005985 NC_005950 NC_006276 NC_005080 NC_001608 NC_003761 NC_003872 NC_007372 NC_007738 NC_004205 NC_007339 NC_005175 NC_006447 NC_004001 NC_003811 NC_002190 NC_006317 NC_005843 NC_007743 NC_006567 NC_003462 NC_006432 NC_001977 NC_004455 NC_007679 NC_003405 NC_001647 NC_007157 NC_005839 NC_003520 NC_006148 NC_001749 NC_003996 NC_001702 NC_005301 NC_004807 NC_001748 NC_006998 NC_001990 NC_001959 NC_007563 NC_004145 NC_005169 NC_004763 NC_003908 NC_004421 NC_005636 NC_005039 NC_001451 NC_003818 NC_006003 NC_003466 NC_007711 NC_004661 NC_003798 NC_003974 NC_004121 NC_001963 NC_007586 NC_002642 NC_003924 NC_003844 NC_006383 NC_003896 NC_003930 NC_003619 NC_004206 NC_004297 NC_006951 NC_003973 NC_007150 NC_003550 NC_006963 NC_007014 NC_002766 NC_007731 NC_002555 NC_006637 NC_007665 NC_003899 NC_002577 NC_001961 NC_002512 NC_007147 NC_001736 NC_005221 NC_005093 NC_006497 NC_007472 NC_004176 NC_001440 NC_001454 NC_002654 NC_003812 NC_005981 NC_003497 NC_001368 NC_006503 NC_006011 NC_004426 NC_004071 NC_005219 NC_006007 NC_007029 NC_004994 NC_006636 NC_006020 NC_001834 NC_003729 NC_004208 NC_003785 NC_003376 NC_006661 NC_007376 NC_007749 NC_006879 NC_006452 NC_007916 NC_011450 NC_003852 NC_002692 NC_007156 NC_002025 NC_003533 NC_005980 NC_006965 NC_003606 NC_001661 NC_007724 NC_006558 NC_006430 NC_003839 NC_003603 NC_001339 NC_006147 NC_007531 NC_003200 NC_006572 NC_001696 NC_007729 NC_003671 NC_005228 NC_001933 NC_007672 NC_003507 NC_002229 NC_004656 NC_003323 NC_001350 NC_001800 NC_002187 NC_007588 NC_001934 NC_004275 NC_003453 NC_002543 NC_001510 NC_004093 NC_003802 NC_001691 NC_007539 NC_003547 NC_006008 NC_007037 NC_001720 NC_003707 NC_005995 NC_003820 NC_006549 NC_003563 NC_001793 NC_003617 NC_003990 NC_004636 NC_001362 NC_007211 NC_005032 NC_003602 NC_003657 NC_001796 NC_002169 NC_003433 NC_001653 NC_004654 NC_004294 NC_003906 NC_003225 NC_005902 NC_007906 NC_006063 NC_003474 NC_001844 NC_005999 NC_007018 NC_003500 NC_003645 NC_004257 NC_003083 NC_007919 NC_004647 NC_003653 NC_003886 NC_005048 NC_003814 NC_007657 NC_005309 NC_001739 NC_004098 NC_003487 NC_004753 NC_003677 NC_003475 NC_002022 NC_003472 NC_002640 NC_001574 NC_007454 NC_006494 NC_002985 NC_005095 NC_003679 NC_004047 NC_002510 NC_003624 NC_002047 NC_007040 NC_004120 NC_003758 NC_005235 NC_006956 NC_003092 NC_004425 NC_002552 NC_005164 NC_003692 NC_002469 NC_007609 NC_003643 NC_006498 NC_005052 NC_007554 NC_007587 NC_005136 NC_007382 NC_002016 NC_006051 NC_001497 NC_007664 NC_000943 NC_003788 NC_003897 NC_001615 NC_003517 NC_001511 NC_001826 NC_005320 NC_001984 NC_002743 NC_004713 NC_003654 NC_005903 NC_005075 NC_004675 NC_007736 NC_005904 NC_005227 NC_007470 NC_007192 NC_004286 NC_007220 NC_004725 NC_007746 NC_004045 NC_003545 NC_007567 NC_001488 NC_001734 NC_001427 NC_003889 NC_006630 NC_005053 NC_003804 NC_003988 NC_005065 NC_004546 NC_006659 NC_003834 NC_003696 NC_003310 NC_004202 NC_003658 NC_007069 NC_003848 NC_003469 NC_006015 NC_001847 NC_001812 NC_001575 NC_003389 NC_006937 NC_007966 NC_001481 NC_001458 NC_002161 NC_001722 NC_004108 NC_001701 NC_007748 NC_007524 NC_004609 NC_003808 NC_003836 NC_001526 NC_004630 NC_005078 NC_005831 NC_003498 NC_007965 NC_007360 NC_005062 NC_007219 NC_003669 NC_006010 NC_002359 NC_006662 NC_007721 NC_001938 NC_005852 NC_001785 NC_007619 NC_002327 NC_004068 NC_003887 NC_002020 NC_003399 NC_004183 NC_007920 NC_001663 NC_001617 NC_005049 NC_005079 NC_003479 NC_003093 NC_001355 NC_003784 NC_005138 NC_001931 NC_002984 NC_004003 NC_004285 NC_007030 NC_003646 NC_001465 NC_003510 NC_005029 NC_006013 NC_003752 NC_003609 NC_001502 NC_003694 NC_004270 NC_005991 NC_005215 NC_006440 NC_001814 NC_004648 NC_001837 NC_004580 NC_004195 NC_007180 NC_006309 NC_003502 NC_004716 NC_003898 NC_003711 NC_000960 NC_001962 NC_007542 NC_001839 NC_003470 NC_003763 NC_002039 NC_007002 NC_003626 NC_003608 NC_005978 NC_003977 NC_001467 NC_005993 NC_006318 NC_003774 NC_004655 NC_005854 NC_005267 NC_007025 NC_003985 NC_003868 NC_004060 NC_002024 NC_001413 NC_007580 NC_005214 NC_005288 NC_005225 NC_005974 NC_003542 NC_003045 NC_004573 NC_006574 NC_007670 NC_002351 NC_000882 NC_001926 NC_005832 NC_001539 NC_004628 NC_007663 NC_002201 NC_005163 NC_000942 NC_003615 NC_002563 NC_007358 NC_004778 NC_001728 NC_006501 NC_001936 NC_003560 NC_001940 NC_005234 NC_007674 NC_003569 NC_003762 NC_001450 NC_001943 NC_006654 NC_007583 NC_001719 NC_007662 NC_004906 NC_004322 NC_003015 NC_003627 NC_006060 NC_003023 NC_007529 NC_003704 NC_004013 NC_001906 NC_002045 NC_001452 NC_002063 NC_003750 NC_005210 NC_003801 NC_003482 NC_004639 NC_001469 NC_007612 NC_006852 NC_003566 NC_003628 NC_006313 NC_004718 NC_005148 NC_003534 NC_001981 NC_001484 NC_007590 NC_003555 NC_001523 NC_001802 NC_004177 NC_004034 NC_001789 NC_007361 NC_001438 NC_001457 NC_003687 NC_004074 NC_006657 NC_001266 NC_007654 NC_001550 NC_004450 NC_006505 NC_001540 NC_005777 NC_007669 NC_001501 NC_002602 NC_006025 NC_003732 NC_007732 NC_005954 NC_003635 NC_005092 NC_001676 NC_005084 NC_003642 NC_007158 NC_001716 NC_003379 NC_007020 NC_004146 NC_007003 NC_005040 NC_005947 NC_002665 NC_007210 NC_005497 NC_001669 NC_007545 NC_005778 NC_001982 NC_003633 NC_003737 NC_003094 NC_004651 NC_001616 NC_003409 NC_001522 NC_001472 NC_004295 NC_004912 NC_002034 NC_005218 NC_007611 NC_007223 NC_004424 NC_004191 NC_006144 NC_007534 NC_007558 NC_002991 NC_004035 NC_001359 NC_003138 NC_007035 NC_005811 NC_003697 NC_003756 NC_002041 NC_005989 NC_007591 NC_002023 NC_003825 NC_004137 NC_001935 NC_005899 NC_005236 NC_003866 NC_001460 NC_001428 NC_006943 NC_004281 NC_006438 NC_003004 NC_005132 NC_006433 NC_003483 NC_006006 NC_005848 NC_005890 NC_003010 NC_004215 NC_002160 NC_004117 NC_003512 NC_002349 NC_005281 NC_001993 NC_005097 NC_003016 NC_003622 NC_004210 NC_005099 NC_003698 NC_003701 NC_004119 NC_006946 NC_004612 NC_005082 NC_007653 NC_006579 NC_004755 NC_007676 NC_003821 NC_001925 NC_004582 NC_005975 NC_003641 NC_007544 NC_002325 NC_004364 NC_001989 NC_007027 NC_003017 NC_004904 NC_001630 NC_001515 NC_005990 NC_003749 NC_004283 NC_004634 NC_007375 NC_001430 NC_004540 NC_003445 NC_002027 NC_007362 NC_006311 NC_007557 NC_004011 NC_007338 NC_003992 NC_001442 NC_001595 NC_006358 NC_003790 NC_006428 NC_006324 NC_004761 NC_007159 NC_001896 NC_007728 NC_003468 NC_004209 NC_003610 NC_006952 NC_006289 NC_003885 NC_005224 NC_004825 NC_004015 NC_007163 NC_001786 NC_004122 NC_003730 NC_006273 NC_007921 NC_004323 NC_003618 NC_006504 NC_003412 NC_007562 NC_002565 NC_006054 NC_004053 NC_001625 NC_005348 NC_002035 NC_003751 NC_001813 NC_003348 NC_005976 NC_007001 NC_003819 NC_007555 NC_007733 NC_002686 NC_005776 NC_004812 NC_004043 NC_007366 NC_004277 NC_004008 NC_001633 NC_004828 NC_003650 NC_004910 NC_001650 NC_006551 NC_006655 NC_002526 NC_007471 NC_004212 NC_005223 NC_001591 NC_005046 NC_003983 NC_004159 NC_001958 NC_004039 NC_003349 NC_007241 NC_004016 NC_002468 NC_004915 NC_005346 NC_006441 NC_006644 NC_007459 NC_003220 NC_003491 NC_001915 NC_003564 NC_003652 NC_002618 NC_003778 NC_004136 NC_002598 NC_004324 NC_006573 NC_003543 NC_003536 NC_001434 NC_002210 NC_005226 NC_004278 NC_005347 NC_005058 NC_005217 NC_005041 NC_001726 NC_003346 NC_001343 NC_002211 NC_003665 NC_006642 NC_007341 NC_004254 NC_001596 NC_003024 NC_004203 NC_003640 NC_004197 NC_003659 NC_003860 NC_004105 NC_007464 NC_001517 NC_002017 NC_005807 NC_000855 NC_004659 NC_004051 NC_007155 NC_007026 NC_003826 NC_007570 NC_006062 NC_004646 NC_000939 NC_005987 NC_001401 NC_007740 NC_003805 NC_004002 NC_003817 NC_001583 NC_003382 NC_001492 NC_002816 NC_004724 NC_003496 NC_006561 NC_003675 NC_006634 NC_004625 NC_003673 NC_002037 NC_007815 NC_005321 NC_003604 NC_007756 NC_003054 NC_003733 NC_003481 NC_003638 NC_002554 NC_004161 NC_001361 NC_003871 NC_002076 NC_007754 NC_003532 NC_003870 NC_006053 NC_003493 NC_003857 NC_003862 NC_004090 NC_000947 NC_001554 NC_006151 NC_001436 NC_002685 NC_003529 NC_001870 NC_007221 NC_004276 NC_007548 NC_002040 NC_003813 NC_001439 NC_004192 NC_001838 NC_004558 NC_007015 NC_007448 NC_006021 NC_003605 NC_007648 NC_007739 NC_004261 NC_005983 NC_003831 NC_007038 NC_001586 NC_004559 NC_005876 NC_004147 NC_007816 NC_004756 NC_003853 NC_004291 NC_007725 NC_007242 NC_004033 NC_007560 NC_006999 NC_003607 NC_003823 NC_003764 NC_005905 NC_001346 NC_003022 NC_004560 NC_006061 NC_007573 NC_006066 NC_003449 NC_003629 NC_004200 NC_004054 NC_001822 NC_003688 NC_007466 NC_003900 NC_005077 NC_002205 NC_005059 NC_003706 NC_001475 NC_005283 NC_001411 NC_005812 NC_004717 NC_004356 NC_002050 NC_007589 NC_001747 NC_003559 NC_007752 NC_001875 NC_002331 NC_002058 NC_006450 NC_004542 NC_003736 NC_007433 NC_006939 NC_003856 NC_001506 NC_003521 NC_004259 NC_007730 NC_004809 NC_003549 NC_003471 NC_003655 NC_001619 NC_003890 NC_004064 NC_004287 NC_006508 NC_010624 NC_005851 NC_007547 NC_005992 NC_003506 NC_004124 NC_005337 NC_006014 NC_006550 NC_006454 NC_001132 NC_005304 NC_006262 NC_006656 NC_001357 NC_001507 NC_003806 NC_006431 NC_001664 NC_001655 NC_002326 NC_006640 NC_001576 NC_007592 NC_006964 NC_001493 NC_003672 NC_007905 NC_001818 NC_001815 NC_004667 NC_002738 NC_004258 NC_003786 NC_006560 NC_004909 NC_004010 NC_001555 NC_001937 NC_002251 NC_002567 NC_004217 NC_002615 NC_002532 NC_001803 NC_007913 NC_006502 NC_003810 NC_003710 NC_001612 NC_003465 NC_003558 NC_007750 NC_003722 NC_001563 NC_005635 NC_005845 NC_007222 NC_007652 NC_006948 NC_007041 NC_003464 NC_002645 NC_004627 NC_003019 NC_001725 NC_007193 NC_004608 NC_004780 NC_001658 NC_001987 NC_005286 NC_006935 NC_006012 NC_005179 NC_007922 NC_003883 NC_002792 NC_006559 NC_004272 NC_003003 NC_000903 NC_001930 NC_004643 NC_006495 NC_005874 NC_007666 NC_007036 NC_006065 NC_004442 NC_003768 NC_004092 NC_006359 NC_003077 NC_007378 NC_003326 NC_004927 NC_006556 NC_007574 NC_003670 NC_004764 NC_007408 NC_003668 NC_005264 NC_003518 NC_004607 NC_007485 NC_002031 NC_004158 NC_003639 NC_004157 NC_005176 NC_001859 NC_004290 NC_001718 NC_003832 NC_007569 NC_004730 NC_004153 NC_004282 NC_001654 NC_002019 NC_003224 NC_007745 NC_002356 NC_007753 NC_003203 NC_006648 NC_004544 NC_006429 NC_005300 NC_003850 NC_003531 NC_004752 NC_003478 NC_001405 NC_005319 NC_001641 NC_002560 NC_003417 NC_007161 NC_005036 NC_001402 NC_003700 NC_005946 NC_004729 NC_006460 NC_007668 NC_006439 NC_003511 NC_002500 NC_001478 NC_004065 NC_003009 NC_003884 NC_005869 NC_003567 NC_006947 NC_007540 NC_002358 NC_004004 NC_006024 NC_003043 NC_006000 NC_002564 NC_000940 NC_001356 NC_003623 NC_007565 NC_006442 NC_003535 NC_006022 NC_003509 NC_005982 NC_006315 NC_002794 NC_005154 NC_007755 NC_005160 NC_005287 NC_003514 NC_001560 NC_001468 NC_001512 NC_003537 NC_002049 NC_003243 NC_007289 NC_005057 NC_006652 NC_002051 NC_003783 NC_003843 NC_007364 NC_004367 NC_003418 NC_001729 NC_003446 NC_003495 NC_006445 NC_001412 NC_006575 NC_006641 NC_003014 NC_005031 NC_005336 NC_002604 NC_001824 NC_004190 NC_002204 NC_003780 NC_004097 NC_004765 NC_005998 NC_003739 NC_001899 NC_004142 NC_001354 NC_007546 NC_004903 NC_007518 NC_006272 NC_003755 NC_003816 NC_005988 NC_003513 NC_001508 NC_003792 NC_002786 NC_003725 NC_005209 NC_004541 NC_004189 NC_006002 NC_006367 NC_005238 NC_001480 NC_004046 NC_001929 NC_005170 NC_007649 NC_003007 NC_004180 NC_005849 NC_002562 NC_004441 NC_005161 NC_004907 NC_005168 NC_001367 NC_005875 NC_001836 NC_006320 NC_007568 NC_001348 NC_004267 NC_001445 NC_007727 NC_001921 NC_001721 NC_005855 NC_006296 NC_005338 NC_006150 NC_003434 NC_004091 NC_007550 NC_005074 NC_004754 NC_004440 NC_003515 NC_005155 NC_001781 NC_001659 NC_007447 NC_001623 NC_004104 NC_005064 NC_003038 NC_003614 NC_007620 NC_001495 NC_006874 NC_005233 NC_006456 NC_004439 NC_004715 NC_003841 NC_004657 NC_004042 NC_007582 NC_003776 NC_006264 NC_001358 NC_001939 NC_006152 NC_001500 NC_007154 NC_002323 NC_003620 NC_004007 NC_006017 NC_001504 NC_003504 NC_002817 NC_006260 NC_002208 NC_006451 NC_001792 NC_003854 NC_003397 NC_005261 NC_006059 NC_003975 NC_005157 NC_006955 NC_004452 NC_003355 NC_003375 NC_003824 NC_001486 NC_003742 NC_002077 NC_005818 NC_003699 NC_003377 NC_001347 NC_001541 NC_005331 NC_006660 NC_006307 NC_003702 NC_005047 NC_002513 NC_005266 NC_006962 NC_007741 NC_004144 NC_004782 NC_003845 NC_004181 NC_003800 NC_007673 NC_003380 NC_004614 NC_001932 NC_003795 NC_005341 NC_004569 NC_003728 NC_007526 NC_005220 NC_004260 NC_006018 NC_003565 NC_002981 NC_001505 NC_003982 NC_002702 NC_004169 NC_004037 NC_003006 NC_003747 NC_003400 NC_005081 NC_002559 NC_004781 NC_007535 NC_004263 NC_002306 NC_004178 NC_007528 NC_003724 NC_002568 NC_003561 NC_004714 NC_003410 NC_004293 NC_002800 NC_003674 NC_007162 NC_005172 NC_004674 NC_005094 NC_001490 NC_003027 NC_005872 NC_001479 NC_003693 NC_003530 NC_002593 NC_003494 NC_004207 NC_007533 NC_006645 NC_001699 NC_007527 NC_002328 NC_007363 NC_003855 NC_007552 NC_006876 NC_003773 NC_001513 NC_007757 NC_001549 NC_003021 NC_002802 NC_003744 NC_007655 NC_003452 NC_003734 NC_005977 NC_007639 NC_001414 NC_004109 NC_003759 NC_004179 NC_003523 NC_007033 NC_007660 NC_004019 NC_001556 NC_003849 NC_006306 NC_001538 NC_006623 NC_003448 NC_004779 NC_003827 NC_001542 NC_006653 NC_004266 NC_004106 NC_003815 NC_003025 NC_007373 NC_005339 NC_001600 NC_006064 NC_001466 NC_004269 NC_001498 NC_003548 NC_007556 NC_003544 NC_003769 NC_004611 NC_007585 NC_002600 NC_000899 NC_003381 NC_001841 NC_006263 NC_001873 NC_001564 NC_004214 NC_001363 NC_004366 NC_005162 NC_006960 NC_006057 NC_007538 NC_005844 NC_001777 NC_006314 NC_007340 NC_004296 NC_004062 NC_003644 NC_001876 NC_002195 NC_006384 NC_005222 NC_004644
