# Supplementary material for: Viral communities associated with healthy and bleaching corals
Source: Environ Microbiol. 2008 Sep;10(9):2277–86. doi: 10.1111/j.1462-2920.2008.01652.x (PMC2702503; doi:10.1111/j.1462-2920.2008.01652.x)
Supplement: Table S7. — Diversity and structure of the viral community in healthy Diploria strigosa tissues. Contig spectrum was tallied from metagenome sequence overlaps and used to predict aspects of viral community structure and diversity using the PHACCS online tool. [file emi0010-2277-SD2.doc]

**Table S1.** Viral particle abundance in raw and homogenized DsH tissue blastate. Viral particles were counted in homogenized and un-homogenized coral blastate using epifluorescence microscopy to determine whether mechanical homogenization resulted in shearing and loss of viral particles.

| Treatment | Control (Not homogenized) | Homogenized (5000 rpm) |
| --- | --- | --- |
| Number of fields analyzed | 6 | 5 |
| Average viral particles per field | 295 | 305.2 |
| Two-sample T-TEST (2-tailed, equal variance): p=0.88 | | |

**Table S2.** TBLASTX hits to virus sequences in GenBank. Sequence libraries DsH and DsB were compared to the GenBank NR database using an E-value cutoff of 0.001. The top virus sequence hit for each metagenome sequence was used to calculate totals. Percentages are calculated from the total hits in each of two categories: sequences from Eukaryote-specific viruses and sequences from phages. Hits to herpesvirus sequences are sorted according to their subfamily within the family *Herpesviridae*; all other virus hits are sorted according to virus family, if such classification exists. Hits to phage sequences are categorized by family according to the Phage Proteomic Tree and further sorted by phage morphology. ND = no classification data available.

| Eukaryote-Specific Viruses | | | DsH Hits | | DsB Hits | |
| --- | --- | --- | --- | --- | --- | --- |
| Family/Subfamily | Genus | Virus name / Common name | No. | % | No. | % |
| Subfamily within family *Herpesviridae* |  |  |  |  |  |  |
| *Alphaherpesvirinae* | *Simplexvirus* | Cercopithecine herpesvirus 2 | 11 | 11% | 7 | 8.2% |
| *Alphaherpesvirinae* | *Simplexvirus* | Cercopithecine herpesvirus 1/Monkey B virus | 6 | 6.1% | 13 | 15% |
| *Alphaherpesvirinae* | *Simplexvirus* | Ateline herpesvirus 3 | 4 | 4.1% | 2 | 2.4% |
| *Alphaherpesvirinae* | *Simplexvirus* | Human herpesvirus 2/Herpes simplex virus type 2 | 3 | 3.1% | 4 | 4.7% |
| *Alphaherpesvirinae* | *Simplexvirus* | Human herpesvirus 1/Herpes simplex virus 1 | 4 | 4.1% |  |  |
| *Alphaherpesvirinae* | *Varicellovirus* | Bovine herpesvirus 5 | 10 | 10% | 8 | 9.4% |
| *Alphaherpesvirinae* | *Varicellovirus* | Suid herpesvirus 1/Pseudorabies virus | 5 | 5.1% | 7 | 8.2% |
| *Alphaherpesvirinae* | *Varicellovirus* | Bovine herpesvirus 1 | 3 | 3.1% | 5 | 5.9% |
| *Alphaherpesvirinae* | *Varicellovirus* | Equid herpesvirus 1/Equine herpesvirus 1 | 1 | 1.0% | 2 | 2.4% |
| *Alphaherpesvirinae* | *Varicellovirus* | Equid herpesvirus 4 | 1 | 1.0% |  |  |
| *Alphaherpesvirinae* | *Varicellovirus* | Human herpesvirus 3/Varicella zoster virus | 1 | 1.0% |  |  |
| *Betaherpesvirinae* | *Cytomegalovirus* | Human herpesvirus 5/Human cytomegalovirus | 1 | 1.0% |  |  |
| *Betaherpesvirinae* | *Muromegalovirus* | Murid herpesvirus 1/Mouse cytomegalovirus 1 | 2 | 2.0% |  |  |
| *Betaherpesvirinae* | *Muromegalovirus* | Murid herpesvirus 2/Rat cytomegalovirus Maastricht |  |  | 2 | 2.4% |
| *Betaherpesvirinae* | Uncl. *Betaherpesvirinae* | Tupaiid herpesvirus 1 | 2 | 2.0% | 1 | 1.2% |
| *Gammaherpesvirinae* | *Rhadinovirus* | Saimiriine herpesvirus 2/Herpesvirus saimiri | 8 | 8.2% | 7 | 8.2% |
| *Gammaherpesvirinae* | *Rhadinovirus* | Human herpesvirus 8/Kaposi's sarcoma-associated HV | 3 | 3.1% | 7 | 8.2% |
| *Gammaherpesvirinae* | *Rhadinovirus* | Equid herpesvirus 2 | 1 | 1.0% |  |  |
| *Gammaherpesvirinae* | *Rhadinovirus* | Alcelaphine herpesvirus 1/Wildebeest herpesvirus | 1 | 1.0% |  |  |
| *Gammaherpesvirinae* | *Lymphocryptovirus* | Human herpesvirus 4/Epstein-Barr virus | 1 | 1.0% | 2 | 2.4% |
| Uncl. *Herpesviridae* | ND | Stealth virus 1 |  |  | 4 | 4.7% |
|  |  | Total hits to herpesviruses | 68 | 69% | 71 | 84% |
| Family  (non-herpesvirus) |  |  |  |  |  |  |
| *Nimaviridae* | *Whispovirus* | Shrimp white spot syndrome virus | 1 | 1.0% |  |  |
| *Baculoviridae* | *Nucleopolyhedrovirus* | *Autographa californica* nucleopolyhedrovirus | 1 | 1.0% |  |  |
| *Baculoviridae* | *Nucleopolyhedrovirus* | *Orgyia pseudotsugata* multicapsid nucleopolyhedrovirus | 1 | 1.0% |  |  |
| *Poxviridae* | *Parapoxvirus* | Bovine papular stomatitis virus | 2 | 2.0% | 3 | 3.5% |
| *Poxviridae* | *Parapoxvirus* | Orf virus/Parapoxvirus ovis | 2 | 2.0% | 1 | 1.2% |
| *Iridoviridae* | *Ranavirus* | Frog virus 3 | 2 | 2.0% |  |  |
| *Iridoviridae* | *Ranavirus* | *Rana tigrina* ranavirus | 1 | 1.0% |  |  |
| *Iridoviridae* | *Ranavirus* | Singapore grouper iridovirus | 1 | 1.0% |  |  |
| *Iridoviridae* | *Lymphocystisvirus* | Lymphocystis disease virus 1 | 1 | 1.0% |  |  |
| *Iridoviridae* | *Coccolithovirus* | Chilo iridescent virus |  |  | 2 | 2.4% |
| *Phycodnaviridae* | *Coccolithovirus* | *Emiliania huxleyi* virus 86 isolate EhV86 | 4 | 4.1% | 4 | 4.7% |
| *Phycodnaviridae* | *Phaeovirus* | *Ectocarpus siliculosus* virus | 1 | 1.0% |  |  |
| *Phycodnaviridae* | *Chlorovirus* | *Paramecium bursaria* Chlorella virus 1 | 3 | 3.1% | 3 | 3.5% |
| *Phycodnaviridae* | *Chlorovirus* | Chlorella virus/*Paramecium bursarium* Chlorella virus | 1 | 1.0% |  |  |
| *Picornaviridae* | *Cardiovirus* | Encephalomyocarditis virus | 1 | 1.0% |  |  |
| *Tymoviridae* | *Maculavirus* | Grapevine fleck virus | 3 | 3.1% | 1 | 1.2% |
| *Retroviridae* | *Alpharetrovirus* | Avian musculoaponeurotic fibrosarcoma virus | 1 | 1.0% |  |  |
| ND | ND | *Pan troglodytes* endogenous retrovirus 1 | 1 | 1.0% |  |  |
| *Retroviridae* | *Mimivirus* | *Acanthamoeba polyphaga* mimivirus | 2 | 2.0% |  |  |
| ND | ND | *Heliothis zea* virus 1 | 1 | 1.0% |  |  |
|  |  | Total hits to Eukaryote-specific non-herpes viruses | 30 | 31% | 14 | 16% |
|  |  |  |  |  |  |  |
|  |  | Total Eukaryote-specific virus hits | 98 |  | 85 |  |

| Phages | | DsH Hits | | DsB Hits | |
| --- | --- | --- | --- | --- | --- |
| Phage Name | Family on Phage Proteomic Tree | # of Hits | % of Total | # of Hits | % of Total |
| Cyanophage P-SSP7 | T7-like podophage | 18 | 9.6% | 41 | 27% |
| Cyanophage P60 | T7-like podophage | 15 | 8.0% | 7 | 4.6% |
| Enterobacteria phage T7 | T7-like podophage | 15 | 8.0% | 1 | 0.7% |
| Roseophage SIO1 | T7-like podophage | 7 | 3.7% | 5 | 3.3% |
| *Pseudomonas aeruginosa* phage PaP3 | T7-like podophage | 4 | 2.1% | 5 | 3.3% |
| Vibriophage VP2 | Unclassified podophage | 4 | 2.1% | 1 | 0.7% |
| *Bordetella* phage BIP-1 | Unclassified podophage | 2 | 1.1% | 0 | 0.0% |
| *Burkholderia cepacia* phage Bcep22 | Unclassified podophage | 2 | 1.1% | 1 | 0.7% |
| Enterobacteria phage epsilon15 | P22-like podophage | 1 | 0.5% | 1 | 0.7% |
| Streptococcus phage C1 | Phi29-like podophage | 1 | 0.5% | 0 | 0.0% |
| Bacteriophage SPP1 | PZA-like podophage | 1 | 0.5% | 1 | 0.7% |
| Enterobacteria phage K1-5 | T7-like podophage | 1 | 0.5% | 0 | 0.0% |
| *Pseudomonas* phage gh-1 | T7-like podophage | 1 | 0.5% | 0 | 0.0% |
| Uncultured T7-like podovirus | T7-like podophage | 1 | 0.5% | 0 | 0.0% |
| Vibriophage VP4 | T7-like podophage | 1 | 0.5% | 0 | 0.0% |
| *Yersinia pestis* phage phiA1122 | T7-like podophage | 1 | 0.5% | 1 | 0.7% |
| *Acyrthosiphon pisum* bacteriophage APSE-1 | Unclassified podophage | 1 | 0.5% | 0 | 0.0% |
| *Acyrthosiphon pisum* bacteriophage APSE-2 | Unclassified podophage | 1 | 0.5% | 0 | 0.0% |
| Podovirus SOG | Unclassified podophage | 1 | 0.5% | 0 | 0.0% |
| Vibriophage VP5 | Unclassified podophage | 1 | 0.5% | 3 | 2.0% |
| *S. typhimurium* bacteriophage ES18 | Unclassified podophage | 1 | 0.5% | 0 | 0.0% |
| Enterobacteria phage P1 | PZA-like podophage | 0 | 0.0% | 2 | 1.3% |
| Bacteriophage T3 | T7-like podophage | 0 | 0.0% | 2 | 1.3% |
| Enterobacteria phage SP6 | T7-like podophage | 0 | 0.0% | 2 | 1.3% |
| Bordtella phage BMP-1 | Unclassified podophage | 0 | 0.0% | 1 | 0.7% |
| Bacteriophage phiKMV | T7-like podophage | 0 | 0.0% | 1 | 0.7% |
| Vibriophage VpV262 | T7-like podophage | 0 | 0.0% | 1 | 0.7% |
| Podovirus GOM | Unclassified podophage | 0 | 0.0% | 1 | 0.7% |
|  | Total podophage | 80 | 43% | 77 | 51% |
|  |  |  |  |  |  |
| Cyanophage P-SSM2 | T4-like myophage | 13 | 6.9% | 13 | 8.6% |
| Bacteriophage KVP40 | T4-like myophage | 4 | 2.1% | 0 | 0.0% |
| Bacteriophage S-PM2 | T4-like myophage | 4 | 2.1% | 0 | 0.0% |
| Cyanophage P-SSM4 | T4-like myophage | 4 | 2.1% | 2 | 1.3% |
| Bacteriophage S-RSM2 | Unclassified myophage | 3 | 1.6% | 1 | 0.7% |
| Mycobacteriophage Bxz1 | Unclassified myophage | 3 | 1.6% | 0 | 0.0% |
| *H. salinarum* virus phiH | PhiH-like myophage | 2 | 1.1% | 1 | 0.7% |
| Bacteriophage Aaphi23 | Unclassified myophage | 2 | 1.1% | 3 | 2.0% |
| Bacteriophage S-WHM1 | Unclassified myophage | 2 | 1.1% | 1 | 0.7% |
| *Burkholderia cenocepacia* phage Bcep1 | Unclassified myophage | 2 | 1.1% | 0 | 0.0% |
| Enterobacteriophage RB43 | T4-like myophage | 2 | 1.1% | 1 | 0.7% |
| *Burkholderia cenocepacia* phage BcepMu | Mu-like myophage | 1 | 0.5% | 0 | 0.0% |
| Enterobacteria phage Mu | Mu-like myophage | 1 | 0.5% | 0 | 0.0% |
| *Vibrio parahaemolyticus* phage VP16C | P2-like myophage | 1 | 0.5% | 1 | 0.7% |
| Bacteriophage Aeh1 | T4-like myophage | 1 | 0.5% | 0 | 0.0% |
| Bacteriophage EJ-1 | Unclassified myophage | 1 | 0.5% | 0 | 0.0% |
| *Burkholderia cepacia* phage Bcep781 | Unclassified myophage | 1 | 0.5% | 0 | 0.0% |
| Cyanophage S-RSM88 | Unclassified myophage | 1 | 0.5% | 0 | 0.0% |
| *Listeria* bacteriophage P100 | Unclassified myophage | 1 | 0.5% | 1 | 0.7% |
| Bacteriophage LP65 | SPO1-like myophage | 0 | 0.0% | 1 | 0.7% |
| Cyanophage S-BnM1 | Unclassified myophage | 0 | 0.0% | 1 | 0.7% |
| Bacteriophage phi CTX | P2-like myophage | 0 | 0.0% | 1 | 0.7% |
| *Haemophilus* phage HP2 | P2-like myophage | 0 | 0.0% | 1 | 0.7% |
| *Vibrio harveyi* bacteriophage VHML | P2-like myophage | 0 | 0.0% | 3 | 2.0% |
| Enterobacteria phage T6 | T4-like myophage | 0 | 0.0% | 1 | 0.7% |
|  | Total myophage | 49 | 26% | 32 | 21% |
|  |  |  |  |  |  |
| Bacteriophage lambda | Lambda-like siphophage | 3 | 1.6% | 0 | 0.0% |
| Bacteriophage phiE125 | Lambda-like siphophage | 3 | 1.6% | 5 | 3.3% |
| Bacteriophage T5 | T5-like siphophage | 3 | 1.6% | 2 | 1.3% |
| *Burkholderia cepacia* phage Bcep176 | Unclassified siphophage | 3 | 1.6% | 2 | 1.3% |
| *Pseudomonas* phage D3 | D3-like siphophage | 2 | 1.1% | 0 | 0.0% |
| Bacteriophage D3112 | Lambda-like siphophage | 2 | 1.1% | 0 | 0.0% |
| Bacteriophage N15 | Lambda-like siphophage | 2 | 1.1% | 1 | 0.7% |
| Enterobacteria phage HK022 | Lambda-like siphophage | 2 | 1.1% | 1 | 0.7% |
| Phage BP-4795 | Lambda-like siphophage | 2 | 1.1% | 0 | 0.0% |
| Bacteriophage phi JL001 | Unclassified siphophage | 2 | 1.1% | 0 | 0.0% |
| Mycobacteriophage CJW1 | Cordog-like siphophage | 1 | 0.5% | 1 | 0.7% |
| Bacteriophage HK97 | Lambda-like siphophage | 1 | 0.5% | 0 | 0.0% |
| Enterobacteria phage P22 | Lambda-like siphophage | 1 | 0.5% | 0 | 0.0% |
| Bacteriophage phiKO2 | Unclassified siphophage | 1 | 0.5% | 0 | 0.0% |
| Mycobacteriophage PG1 | Unclassified siphophage | 1 | 0.5% | 0 | 0.0% |
| Bacteriophage Mx8 | Cordog-like siphophage | 0 | 0.0% | 2 | 1.3% |
| Mycobacteriophage Che8 | Cordog-like siphophage | 0 | 0.0% | 1 | 0.7% |
| Mycobacteriophage TM4 | Cordog-like siphophage | 0 | 0.0% | 3 | 2.0% |
| Bacteriophage bIL285 | D3-like siphophage | 0 | 0.0% | 1 | 0.7% |
| Bacteriophage phi3626 | D3-like siphophage | 0 | 0.0% | 1 | 0.7% |
| *Shigella flexneri* bacteriophage V | D3-like siphophage | 0 | 0.0% | 1 | 0.7% |
| Bacteriophage phi1026b | Lambda-like siphophage | 0 | 0.0% | 1 | 0.7% |
| Bacteriophage PY54 | Unclassified siphophage | 0 | 0.0% | 1 | 0.7% |
| *Methanobacterium* phage psiM2 | Unclassified siphophage | 0 | 0.0% | 1 | 0.7% |
|  | Total siphophage | 29 | 15% | 24 | 16% |
|  |  |  |  |  |  |
| *Actinoplanes* phage phiAsp2 | Unclassified | 4 | 2.1% | 1 | 0.7% |
| uncultured cyanophage | Unclassified | 3 | 1.6% | 1 | 0.7% |
| Bacteriophage VP882 | Unclassified | 3 | 1.6% | 2 | 1.3% |
| Bacteriophage CP-1639 | Unclassified | 2 | 1.1% | 0 | 0.0% |
| Bacteriophage WO | Unclassified | 2 | 1.1% | 2 | 1.3% |
| Bacteriophage WOcauB1 | Unclassified | 2 | 1.1% | 1 | 0.7% |
| *Bacillus clarkii* bacteriophage BCJA1c | Unclassified | 1 | 0.5% | 0 | 0.0% |
| Bacteriophage Rho11s | Unclassified | 1 | 0.5% | 0 | 0.0% |
| Coliphage K1F | Unclassified | 1 | 0.5% | 1 | 0.7% |
| *Mycobacterium* phage DS6A | Unclassified | 1 | 0.5% | 0 | 0.0% |
| *Neisseria meningitidis* phage 2120 | Unclassified | 1 | 0.5% | 1 | 0.7% |
| *Lactobacillus reuteri* phage | Unclassified | 1 | 0.5% | 0 | 0.0% |
| *Wolbachia* Wkue bacteriophage | Unclassified | 0 | 0.0% | 1 | 0.7% |
| Uncultured cyanophage clone Bac9 D04 | Unclassified | 1 | 0.5% | 1 | 0.7% |
| *Burkholderia cepacia* complex phage BcepC6B | Unclassified | 2 | 1.1% | 3 | 2.0% |
| Bacteriophage 187 | Unclassified | 1 | 0.5% | 0 | 0.0% |
| Bacteriophage Felix 01 | Unclassified | 1 | 0.5% | 1 | 0.7% |
| Bacteriophage KS7 | Unclassified | 1 | 0.5% | 0 | 0.0% |
| *Sinorhizobium meliloti* phage PBC5 | Unclassified | 1 | 0.5% | 0 | 0.0% |
| *Xanthomonas campestris* pv. *Pelargonii* phage Xp15 | Unclassified | 1 | 0.5% | 0 | 0.0% |
| Bacteriophage 16-3 | Unclassified | 0 | 0.0% | 1 | 0.7% |
| *Xanthomonas oryzae* bacteriophage Xp10 | Unclassified | 0 | 0.0% | 1 | 0.7% |
|  | Total unclassified phage | 30 | 16% | 17 | 11% |
|  |  |  |  |  |  |
| Bacteriophage SfX | Unclassified inophage | 0 | 0.0% | 1 | 0.7% |
|  | Total phage hits | 188 |  | 151 |  |

**Table S3**. BLASTX hits to herpesvirus genes. Sequences were compared to a database of all complete genomes from Eukaryote-specific viruses. E-value cutoff = 0.001. Only the top hit for each metagenome sequence is listed.

| Metagenome Sequence | Similar To | Herpesvirus Genome | Function | Alternate Function | Percent Identity | Hit Length | Gaps | E-value |
| --- | --- | --- | --- | --- | --- | --- | --- | --- |
| DSH_001_C05 | YP_656628.1 | Ranid herpesvirus 2 | DNA (cytosine-5-)-methyltransferase | similar to RaHV-1 ORF86 | 36.36 | 55 | 0 | 5.00E-05 |
| DSH_002_B07 | YP_001033968.1 | Gallid herpesvirus 2 | ribonucleotide reductase subunit 1 | none listed | 26.9 | 145 | 3 | 2.00E-06 |
| DSH_003_E10 | YP_001096176.1 | Koi herpesvirus | ORF141 | ribonucleotide reductase large subunit | 26.6 | 188 | 4 | 2.00E-11 |
| DSH_008_C12 | YP_067966.1 | Cercopithecine herpesvirus 15 | EBNA-3B | similar to Epstein-Barr virus EBNA-3B (nuclear antigen) | 28.16 | 103 | 1 | 5.00E-04 |
| DSH_012_D02 | YP_001096176.1 | Koi herpesvirus | ORF141 | ribonucleotide reductase large subunit | 29.47 | 95 | 3 | 2.00E-04 |
| DSH_012_D05 | YP_001096176.1 | Koi herpesvirus | ORF141 | ribonucleotide reductase large subunit | 33.68 | 95 | 4 | 6.00E-06 |
| DSH_013_E08 | YP_068003.1 | Cercopithecine herpesvirus 15 | LF3 | similar to Epstein-Barr virus LF3 | 35.09 | 114 | 6 | 2.00E-04 |
| DSH_014_H08 | NP_570820.1 | Cercopithecine herpesvirus 17, genome. | latent nuclear antigen | ORF 73; similar to Kaposi's sarcoma-associated herpesvirus ORF 73 | 36.17 | 94 | 4 | 3.00E-04 |
| DSH_025_A07 | YP_001129431.1 | Human herpesvirus 8 | LANA | latent nuclear antigen; component of latency-associated nuclear antigen; LANA; LNA; ORF73 | 28.7 | 108 | 2 | 2.00E-06 |
| DSB_002_J20 | NP_044888.1 | Murid herpesvirus 4 | glycoprotein 150 | none listed | 30.11 | 186 | 8 | 6.00E-04 |
| DSB_002_E20 | YP_001096184.1 | Koi herpesvirus | ORF149 | predicted membrane glycoprotein; member of ORF25 gene family | 30.87 | 149 | 5 | 8.00E-06 |
| DSB_003_D05 | YP_001096103.1 | Koi herpesvirus | ORF68 | similar to myosin and related proteins, and also to IcHV-1 ORF22 | 23.4 | 141 | 3 | 2.00E-04 |
| DSB_002_M07 | NP_045288.1 | Equid herpesvirus 4 | envelope glycoprotein J | type 1 membrane protein; contains a signal peptide | 26.27 | 217 | 1 | 4.00E-09 |
| DSB_001_G04 | YP_067991.1 | Cercopithecine herpesvirus 15 | BDLF3 | similar to Epstein-Barr virus BDLF3 (glycoprotein gp150) | 27.33 | 161 | 3 | 6.00E-06 |
| DSB_002_F04 | YP_656634.1 | Ranid herpesvirus 2 | ORF126 | similar to RaHV-1 ORF56 family; similar to RaHV-1 ORF89 and IcHV-1 ORF22; contains myosin-like domain | 19.43 | 283 | 8 | 8.00E-04 |
| DSB_001_O03 | YP_001129431.1 | Human herpesvirus 8 | LANA | latent nuclear antigen; component of latency-associated nuclear antigen; LANA; LNA; ORF73 | 25.19 | 131 | 2 | 1.00E-04 |
| DSB_003_L19 | YP_001033965.1 | Gallid herpesvirus 2 | large tegument protein | complexed with tegument protein UL37; ubiquitin-specific protease (N-terminal region) | 36.62 | 71 | 2 | 2.00E-04 |

**Table S4.** BLASTX hits to genes from algae viruses (family *Phycodnaviridae*). Sequences were compared to a database of all complete, annotated genomes from Eukaryote-specific viruses. E-value cutoff = 0.001. Only the top hit for each metagenome sequence is listed.

| Metagenome Sequence | Similar To | Virus Genome | Gene Function | Alternate Function | Percent Identity | Hit Length | Gaps | E-value |
| --- | --- | --- | --- | --- | --- | --- | --- | --- |
| DSH_002_E02 | YP_001427299.1 | Chlorella virus ATCV-1 | hypothetical protein | thymidylate synthase X | 66.0 | 47 | 0 | 2.00E-14 |
| DSH_002_H06 | YP_001426874.1 | Chlorella virus ATCV-1 | hypothetical protein |  | 31.6 | 57 | 0 | 5.00E-04 |
| DSH_008_H02 | YP_001427299.1 | Chlorella virus ATCV-1 | hypothetical protein | thymidylate synthase X | 47.8 | 90 | 2 | 5.00E-16 |
| DSH_009_F11 | YP_001427319.1 | Chlorella virus ATCV-1 | hypothetical protein | ribonucleoside-triphosphate reductase | 34.2 | 111 | 2 | 1.00E-10 |
| DSH_011_E06 | YP_001427319.1 | Chlorella virus ATCV-1 | hypothetical protein | ribonucleoside-triphosphate reductase | 65.5 | 84 | 0 | 2.00E-24 |
| DSH_011_F10 | YP_001427028.1 | Chlorella virus ATCV-1 | hypothetical protein |  | 50 | 60 | 1 | 1.00E-11 |
| DSH_012_D02 | YP_001426582.1 | Chlorella virus ATCV-1 | hypothetical protein | ribonucleotide reductase (large subunit) | 25.2 | 119 | 3 | 6.00E-07 |
| DSH_013_F11 | YP_001427028.1 | Chlorella virus ATCV-1 | hypothetical protein |  | 38.7 | 62 | 2 | 2.00E-07 |
| DSH_014_A06 | YP_001427028.1 | Chlorella virus ATCV-1 | hypothetical protein |  | 51.7 | 60 | 2 | 2.00E-08 |
| DSH_017_B09 | YP_001427299.1 | Chlorella virus ATCV-1 | hypothetical protein | thymidylate synthase X | 31.9 | 119 | 2 | 2.00E-09 |
| DSH_022_E11 | YP_001426582.1 | Chlorella virus ATCV-1 | hypothetical protein | ribonucleotide reductase (large subunit) | 34.2 | 120 | 0 | 4.00E-09 |
| DSH_025_F12 | YP_001426761.1 | Chlorella virus ATCV-1 | hypothetical protein |  | 66.7 | 27 | 0 | 1.00E-04 |
| DSH_002_B02 | YP_001381854.1 | Chlorella virus FR483 | hypothetical protein N277L | putative SWI/SNF helicase | 29.8 | 124 | 2 | 8.00E-05 |
| DSH_009_G12 | YP_001381961.1 | Chlorella virus FR483 | hypothetical protein N535L |  | 27.7 | 202 | 7 | 4.00E-05 |
| DSH_015_C04 | YP_001381961.1 | Chlorella virus FR483 | hypothetical protein N535L |  | 35.7 | 112 | 2 | 7.00E-09 |
| DSH_002_D10 | YP_001381963.1 | Chlorella virus FR483 | hypothetical protein N542L |  | 50 | 30 | 0 | 5.00E-04 |
| DSH_001_A07 | YP_001381971.1 | Chlorella virus FR483 | hypothetical protein N565L |  | 31.3 | 112 | 3 | 4.00E-04 |
| DSH_003_E10 | YP_001382055.1 | Chlorella virus FR483 | hypothetical protein N766L | putative large subunit of ribonucleotide reductase | 30 | 207 | 4 | 4.00E-19 |
| DSH_013_E01 | NP_077667.1 | *Ectocarpus siliculosus* virus | EsV-1-182 | replication factor C small subunit | 34.6 | 110 | 1 | 4.00E-11 |
| DSH_012_D05 | YP_294186.1 | *Emiliania huxleyi* virus 86 | putative ribonucleoside-diphosphate reductase protein | Similar to the N-terminal region of Homo sapiens ribonucleoside-diphosphate reductase M1 chain | 29.9 | 87 | 1 | 3.00E-07 |
| DSH_002_B07 | NP_048985.1 | *Paramecium bursaria* Chlorella virus 1 | hypothetical protein | similar to *Schizosaccharomyces* ribonucleotide reductase M1 chain | 26.9 | 134 | 2 | 2.00E-06 |
| DSH_004_A05 | NP_049030.1 | *Paramecium bursaria* Chlorella virus 1 | hypothetical protein | similar to *Synechocystis* ORF s111635 | 52.8 | 178 | 1 | 1.00E-44 |
| DSH_009_D05 | NP_049030.1 | *Paramecium bursaria* Chlorella virus 1 | hypothetical protein | similar to *Synechocystis* ORF s111635 | 63.6 | 33 | 0 | 2.00E-05 |
| DSH_013_A11 | NP_048466.1 | *Paramecium bursaria* Chlorella virus 1 | hypothetical protein | PBCV-1 GDP-D-mannose dehydratase | 36.2 | 58 | 0 | 6.00E-04 |
| DSH_014_F05 | NP_048711.1 | *Paramecium bursaria* Chlorella virus 1 | hypothetical protein |  | 29.47 | 95 | 3 | 1.00E-04 |
| DSH_021_B01 | NP_048933.1 | *Paramecium bursaria* Chlorella virus 1 | hypothetical protein |  | 45 | 40 | 2 | 8.00E-04 |
| DSH_021_G03 | NP_048532.1 | *Paramecium bursaria* Chlorella virus 1 | hypothetical protein | PBVC-1 DNA polymerase | 34.1 | 88 | 2 | 2.00E-06 |
| DSH_024_H01 | NP_048758.1 | *Paramecium bursaria* Chlorella virus 1 | hypothetical protein |  | 34.8 | 155 | 4 | 5.00E-20 |
| DSH_025_A06 | NP_048779.1 | *Paramecium bursaria* Chlorella virus 1 | hypothetical protein | similar to Bacteriophage SP01 gene 31 intron | 42.6 | 61 | 1 | 5.00E-08 |
| DSH_026_A12 | NP_049013.1 | *Paramecium bursaria* Chlorella virus 1 | hypothetical protein | GDQGE (5x) | 59.4 | 32 | 0 | 1.00E-05 |
| DSB_001_A04 | YP_001426973.1 | Chlorella virus ATCV-1 | hypothetical protein |  | 33.3 | 219 | 7 | 5.00E-13 |
| DSB_001_O09 | YP_001426973.1 | Chlorella virus ATCV-1 | hypothetical protein |  | 33.5 | 212 | 5 | 3.00E-14 |
| DSB_002_L03 | YP_001427319.1 | Chlorella virus ATCV-1 | hypothetical protein | ribonucleoside-triphosphate reductase | 51.9 | 185 | 4 | 3.00E-53 |
| DSB_002_O10 | YP_001427319.1 | Chlorella virus ATCV-1 | hypothetical protein | ribonucleoside-triphosphate reductase | 52.7 | 201 | 3 | 1.00E-52 |
| DSB_003_A01 | YP_001426596.1 | Chlorella virus ATCV-1 | hypothetical protein |  | 40.1 | 142 | 4 | 3.00E-16 |
| DSB_003_C09 | YP_001427148.1 | Chlorella virus ATCV-1 | hypothetical protein | glycosyltransferase | 27.3 | 161 | 5 | 9.00E-07 |
| DSB_003_C13 | YP_001427299.1 | Chlorella virus ATCV-1 | hypothetical protein | thymidylate synthase X | 28 | 143 | 2 | 5.00E-10 |
| DSB_003_E09 | YP_001426668.1 | Chlorella virus ATCV-1 | hypothetical protein | ATP-dependent DNA ligase | 26.8 | 153 | 5 | 4.00E-04 |
| DSB_003_H14 | YP_001426973.1 | Chlorella virus ATCV-1 | hypothetical protein |  | 36.1 | 72 | 1 | 2.00E-04 |
| DSB_003_I04 | YP_001427299.1 | Chlorella virus ATCV-1 | hypothetical protein | thymidylate synthase X | 53 | 168 | 0 | 2.00E-47 |
| DSB_003_K15 | YP_001427028.1 | Chlorella virus ATCV-1 | hypothetical protein |  | 51.6 | 62 | 1 | 7.00E-13 |
| DSB_003_O06 | YP_001427319.1 | Chlorella virus ATCV-1 | hypothetical protein | ribonucleoside-triphosphate reductase | 56.1 | 230 | 2 | 8.00E-68 |
| DSB_001_M11 | YP_001381961.1 | Chlorella virus FR483 | hypothetical protein N535L |  | 25.1 | 199 | 9 | 1.00E-04 |
| DSB_002_N03 | NP_077550.1 | *Ectocarpus siliculosus* virus | EsV-1-65 | viral hybrid histidine kinase | 34.6 | 179 | 5 | 1.00E-17 |
| DSB_002_E20 | YP_294122.1 | *Emiliania huxleyi* virus 86 | hypothetical protein | contains database matches in repeat regions to several proline-rich proteins, for example,*Chlamydomonasincerta* SAG1 plus agglutinin | 30.9 | 139 | 4 | 3.00E-04 |
| DSB_001_G04 | NP_048366.1 | *Paramecium bursaria* Chlorella virus 1 | hypothetical protein | Asn/Thr/Ser/Ile rich protein; similar to *Rickettsia* cell surface antigen | 25.9 | 135 | 5 | 1.00E-04 |
| DSB_001_K15 | NP_048448.1 | *Paramecium bursaria* Chlorella virus 1 | hypothetical protein | PBCV-1 glucosamine synthetase | 31.9 | 72 | 2 | 4.00E-04 |
| DSB_002_D15 | NP_048952.1 | *Paramecium bursaria* Chlorella virus 1 | hypothetical protein | similar to *Vibrio fischeri* dCMP deaminase | 38.6 | 140 | 4 | 2.00E-23 |
| DSB_002_H01 | NP_048466.1 | *Paramecium bursaria* Chlorella virus 1 | hypothetical protein | PBCV-1 GDP-D-mannose dehydratase | 29.8 | 181 | 4 | 6.00E-14 |
| DSB_002_M07 | NP_048362.1 | *Paramecium bursaria* Chlorella virus 1 | hypothetical protein | Asn/Thr/Ser/Val rich protein | 22.8 | 202 | 5 | 7.00E-04 |
| DSB_003_G06 | NP_048904.1 | *Paramecium bursaria* Chlorella virus 1 | hypothetical protein | similar to *Caenorhabditis* transcription activator | 34.6 | 81 | 0 | 7.00E-08 |
| DSB_003_L19 | NP_048405.1 | *Paramecium bursaria* Chlorella virus 1 | hypothetical protein | contains Pro-rich Px motifs: SPKPP (20X), PEPPA (9X) | 43.3 | 60 | 2 | 3.00E-05 |

**Table S5. BLASTX hits to cyanophage genes in the Phage Sequence Databank. (http://phage.sdsu.edu/phage; E-value < 0.001). All metagenome sequences with best hits to cyanophage genes were compiled. Listed are the 15 genes with the lowest E-value in each search and any gene with 4 or more hits. BLASTX statistics are listed for the best hit to each gene. Hits to hypothetical and unknown targets are not shown.**

|  |  | DsH Hits | | | |
| --- | --- | --- | --- | --- | --- |
| Cyanophage Genome | Gene Function | Top Hit % ID | Top Hit Length | Top Hit E-value | # Hits total |
| P-SSP7 | DNA maturase beta subunit | 78.1 | 169 | 8.00E-73 | 3 |
| P60 | DNA polymerase | 40.4 | 166 | 9.00E-31 | 5 |
| P-SSM2 | glycosyltransferase family 25 | 66.9 | 151 | 3.00E-63 | 1 |
| P-SSM2 | glycosyltransferase family 6 | 77.8 | 135 | 4.00E-62 | 1 |
| P-SSM2 | NrdA | 93.0 | 129 | 7.00E-56 | 4 |
| P-SSM4 | NrdA | 90.6 | 127 | 3.00E-53 | 3 |
| P-SSM2 | NrdB | 66.7 | 165 | 1.00E-54 | 1 |
| P-SSM2 | phage tail fiber-like protein | 50.6 | 158 | 4.00E-37 | 10 |
| S-PM2 | photosystem II D1 protein | 94.8 | 134 | 2.00E-67 | 4 |
| P-SSM2 | possible endonuclease | 46.0 | 100 | 2.00E-20 | 9 |
| P-SSM2 | PurM | 85.4 | 123 | 2.00E-60 | 1 |
| P-SSM2 | RegA | 78.1 | 128 | 2.00E-57 | 1 |
| P-SSP7 | ribonucleotide reductase domain | 71.8 | 149 | 7.00E-61 | 8 |
| P-SSM2 | T4-like tail sheath protein | 74.2 | 128 | 7.00E-50 | 3 |
| P-SSP7 | T7-like capsid protein | 71.3 | 143 | 1.00E-57 | 3 |
| P-SSP7 | T7-like head-to-tail connector | 69.7 | 201 | 1.00E-78 | 6 |
| P-SSP7 | T7-like internal core protein | 57.0 | 100 | 4.00E-27 | 4 |
| P-SSP7 | T7-like primase/helicase | 69.1 | 220 | 5.00E-86 | 5 |
| P-SSP7 | T7-like tail tubular protein B | 39.5 | 205 | 2.00E-35 | 7 |
| S-PM2 | thymidilate synthase | 55.6 | 180 | 4.00E-51 | 5 |
|  |  | DsB Hits | | | |
| P-SSM2 | core ps II reaction center protein | 91.6 | 214 | 2.00E-111 | 1 |
| P-SSP7 | DNA maturase beta subunit | 88.9 | 260 | 7.00E-134 | 7 |
| P60 | DNA polymerase | 44.3 | 185 | 4.00E-38 | 5 |
| P-SSM4 | fiber | 29.9 | 204 | 1.00E-10 | 5 |
| P-SSM2 | phage tail fiber-like protein | 38.2 | 212 | 7.00E-29 | 9 |
| S-PM2 | photosystem II D1 protein | 90.4 | 177 | 6.00E-92 | 1 |
| P-SSP7 | ribonucleotide reductase domain | 73.7 | 167 | 4.00E-75 | 6 |
| P-SSM2 | T4-like capsid assembly protein | 67.9 | 237 | 3.00E-96 | 2 |
| P-SSM2 | T4-like DNA pkg lg subunit terminase | 55.5 | 220 | 6.00E-69 | 1 |
| P-SSM2 | T4-like major capsid protein | 65.2 | 276 | 5.00E-88 | 2 |
| P-SSP7 | T7-like capsid protein | 69.6 | 250 | 1.00E-91 | 4 |
| P-SSP7 | T7-like exonuclease | 66.0 | 156 | 3.00E-59 | 4 |
| P-SSP7 | T7-like head-to-tail connector | 63.5 | 271 | 5.00E-96 | 4 |
| P-SSP7 | T7-like internal core protein | 47.2 | 263 | 2.00E-61 | 5 |
| P-SSP7 | T7-like RNA polymerase | 70.2 | 238 | 8.00E-90 | 7 |
| P-SSP7 | T7-like ssDNA binding protein | 56.4 | 188 | 3.00E-54 | 2 |
| P-SSP7 | T7-like tail fiber | 43.3 | 231 | 7.00E-39 | 9 |
| P-SSP7 | T7-like tail tubular protein B | 44.1 | 229 | 3.00E-59 | 6 |

**Table S6. BLASTX hits to vibriophage genes. Sequence libraries were compared to the Phage Sequence Databank using an E-value cutoff of 0.001. All metagenome sequences with best hits to vibriophage genes were compiled. Listed are the 10 genes with the lowest E-value in each search, as well as any gene with 3 or more hits. BLASTX statistics are listed for the best hit to each gene. Hits to hypothetical and unknown targets are not shown.**

|  |  | DsH Hits |  |  |  |
| --- | --- | --- | --- | --- | --- |
| Vibriophage Genome | Gene Function | Top Hit % ID | Top Hit Length | Top Hit E-value | # Hits total |
| KVP40 | aerobic ribonucleoside diphosphate reductase large subunit | 50.0 | 124 | 2.00E-26 | 4 |
| VP4 | DNA packaging protein B | 55.1 | 207 | 5.00E-57 | 4 |
| VP4 | DNA polymerase | 35.3 | 170 | 8.00E-19 | 4 |
| VP4 | DNA primase/helicase | 39.2 | 189 | 4.00E-29 | 5 |
| KVP40 | gp44 | 65.8 | 38 | 1.00E-07 | 3 |
| VP4 | head-to-tail joining protein | 36.0 | 211 | 2.00E-29 | 3 |
| KVP40 | NMN adenylyl tranferase | 30.2 | 265 | 3.00E-25 | 1 |
| VHML | ORF22 | 37.3 | 225 | 1.00E-34 | 1 |
| VP16C | putative tail protein | 49.3 | 201 | 1.00E-46 | 1 |
| KVP40 | RegA | 50.4 | 119 | 3.00E-30 | 1 |
| VP2 | superfamily II DNA/RNA helicase | 39.7 | 174 | 5.00E-29 | 4 |
| KVP40 | tail sheath protein | 61.4 | 132 | 2.00E-41 | 2 |
| VP4 | tail tubular protein B | 32.7 | 98 | 2.00E-08 | 4 |
|  |  | DsB Hits |  |  |  |
| Vibrophage Genome | Function | Top Hit % ID | Top Hit Length | Top Hit E-value | # Hits total |
| VP4 | DNA packaging protein B | 51.5 | 266 | 6.00E-66 | 9 |
| VP4 | DNA polymerase | 28.4 | 271 | 8.00E-21 | 5 |
| VP4 | endonuclease | 52.1 | 119 | 2.00E-26 | 2 |
| VP4 | exonuclease | 38.6 | 145 | 1.00E-22 | 5 |
| KVP40 | gp23 | 45.1 | 213 | 1.00E-41 | 2 |
| VP4 | head-to-tail joining protein | 38.1 | 278 | 1.00E-46 | 4 |
| VHML | ORF22 | 55.1 | 176 | 4.00E-54 | 5 |
| VHML | ORF23 | 32.6 | 239 | 1.00E-23 | 2 |
| KVP40 | portal vertex protein of head | 45.1 | 235 | 1.00E-52 | 2 |
| VP4 | RNA polymerase | 31.8 | 277 | 2.00E-25 | 8 |
| VP4 | tail tubular protein B | 31.8 | 214 | 1.00E-24 | 3 |
| VP2 | terminase | 32.7 | 153 | 1.00E-15 | 3 |
| KVP40 | terminase DNA packaging enzyme large subunit | 40.9 | 220 | 2.00E-42 | 1 |

**Table S7. Diversity and structure of the viral community in healthy *Diploria strigosa* tissues. Contig spectrum was tallied from metagenome sequence overlaps and used to predict aspects of viral community structure and diversity using the PHACCS online tool.**

| Sample – Health | DsH – Healthy |
| --- | --- |
| Number of sequences | 1580 |
| Contig spectrum | 1523 22 3 1 0 0 |
| Richness | 28600 |
| Evenness | 0.873 |
| Most Abundant Genotype | 2.63% |
| Shannon-Weiner Index | 8.96 |
| Average sequence length (bp) | 461 |
